# Additive Engineering for Stable and Efficient Dion–Jacobson Phase Perovskite Solar Cells

**DOI:** 10.1007/s40820-023-01110-9

**Published:** 2023-05-24

**Authors:** Min Liu, Thierry Pauporté

**Affiliations:** Institut de Recherche de Chimie Paris (IRCP), UMR8247, Chimie ParisTech, PSL University, CNRS, 11 Rue P. Et M. Curie, 75005 Paris, France

**Keywords:** Dion–Jacobson phases, Perovskite solar cells, Additive compounds, Defect passivation, Stability

## Abstract

Because of their better chemical stability and fascinating anisotropic characteristics, Dion–Jacobson (DJ)-layered halide perovskites, which owe crystallographic two-dimensional structures, have fascinated growing attention for solar devices. DJ-layered halide perovskites have special structural and photoelectronic features that allow the van der Waals gap to be eliminated or reduced. DJ-layered halide perovskites have improved photophysical characteristics, resulting in improved photovoltaic performance. Nevertheless, owing to the nature of the solution procedure and the fast crystal development of DJ perovskite thin layers, the precursor compositions and processing circumstances can cause a variety of defects to occur. The application of additives can impact DJ perovskite crystallization and film generation, trap passivation in the bulk and/or at the surface, interface structure, and energetic tuning. This study discusses recent developments in additive engineering for DJ multilayer halide perovskite film production. Several additive-assisted bulk and interface optimization methodologies are summarized. Lastly, an overview of research developments in additive engineering in the production of DJ-layered halide perovskite solar cells is offered.

## Highlights

- Soluble compounds are added to the Dion–Jacobson (DJ) perovskite precursor solutions.
- Current studies and development trends of additive compounds in DJ-phase perovskite solar cells are reviewed.
- The innate functions of additive compounds in DJ-phase perovskite solar cells are developed.
- An insightful perspective is outlined for future research in additive compounds for DJ-phase perovskite solar cells.

## Graphical abstract

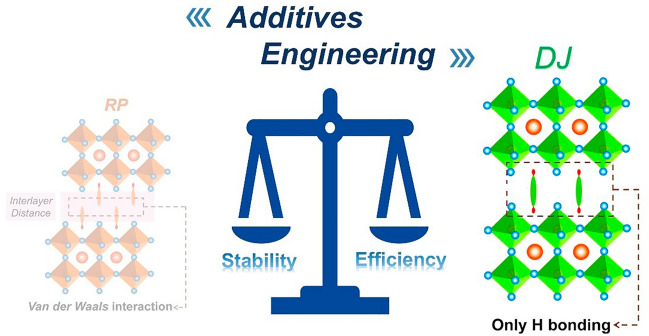

## Introduction

Halide perovskites have acted well in outstanding solar cells which achieved a best efficiency of 25.7% [1–5] and are driving current photovoltaic technology development [6–8]. Nevertheless, the weak stability of halide perovskites stays an essential problem that has to be addressed before this low-priced and solution-processable photovoltaic equipment can be commercialized on a large scale [9, 10].

Prior research has shown that adding large or long-chain monovalent organic cations [11], for example C_4_H_9_NH_3_^+^ [12–15], to create two-dimensional (2D) Ruddlesden–Popper (RP)-layered halide perovskite phases [11, 16] is a useful method for boosting the chemical steadiness of halide perovskites (PVK). Even though these materials appear to have better chemical stability in a variety of environmental settings [5, 17], their photovoltaic performance is typically worse than that of three-dimensional (3D) halide PVK [18, 19], primarily because the intrinsic van der Waals gap hinders carrier transport across the long organic spacers [20–23]. To attain improved steadiness although limiting carrier transport in solar cells, it is important to look into strategies to offset the unfavorable impacts of such interlayer constructions as those observed in RP-layered halide perovskites [24–27].

**Fig. 1:**
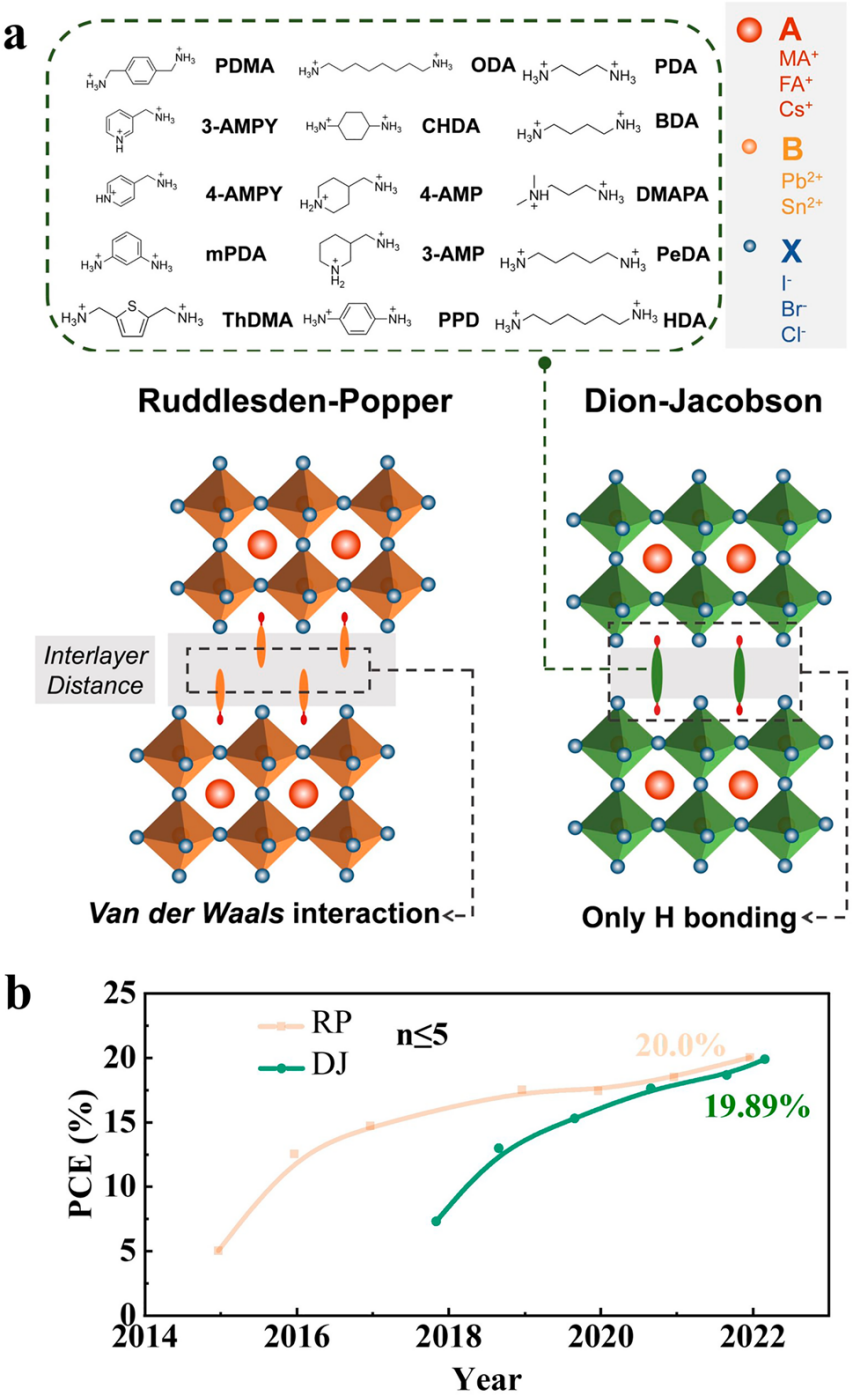
**a** DJ and RP phase structural comparison. The most often used DJ interlayer cations are: 1,3-propanediammonium (PDA), 1,4-butanediammonium (BDA), 3-(dimethylammonium)-1-propylammonium (DMAPA), 1,5-pentamethylenediammonium (PeDA), 1,6-hexamethylenediammonium (HDA), 1,8-octanediammonium (ODA), trans-1,4-cyclohexanediammonium (CHDA), 4-(aminomethyl)piperidinium (4-AMP), 3-(aminomethyl)piperidinium (3-AMP), p-phenylenediammonium (PPD), 1,4-phenylenedimethaneammonium (PDMA), 3-(aminomethyl)pyridinium (3-AMPY), 4-(aminomethyl)pyridinium (4-AMPY), m-phenylenediammonium (mPDA), 2,5-thiophenedimethylammonium (ThDMA) [50]. Copyright 2022, Wiley–VCH. **b** The best PCE evolution for RP and DJ PSCs for n ≤ 5 [51, 52]

Dion–Jacobson (DJ) phase-layered halide PVK are the subject of this review since they have received a lot of interest from scientists. DJ-layered halide perovskites have the chemical formula (*A*’)(*A*)_*n*−1_*B*_*n*_*X*_3*n*+1_ (*A* = CH_3_NH_3_^+^ (MA^+^), HC(NH_2_)_2_^+^ (FA^+^), and/or Cs^+^; *B* = Sn^2+^ and/or Pb^2+^, *X* = Cl^−^, Br^−^ and/or) *I*^−^ [28, 29], which is a derivation of ABX_3_ [30–34]. Divalent interlayer organic cations in structure *A*' are what set it apart [35, 36]. In Fig. 1a, the potential chemical components of *A*' interlayer cations are shown [37]. As schematically shown in Fig. 1a, these innovative chemical configurations of organic cation interlayers reduce the interlayer spacing and delete the van der Waals gap in DJ phases [38]. These distinct interlayer chemistry designs encourage increased stability while opening a higher likelihood of carrier hopping or tunnelling [39]. As a result, the potential of DJ-stacked halide perovskite to provide more perfect device performance in solar cells has been proven [40, 41].

To enhance DJ PSC performance, several strategies have been proposed [40, 42, 43]. Among these, additives are often employed and have been crucial to several discoveries [44, 45]. In addition to increasing the DJ PSCs' efficiency, additives are also used to reduce hysteresis and boost stability [46]. The hysteresis is affected by the interaction of ion movement, charge buildup, and charge trapping/detrapping. Here, a significant portion is attributable to the movement of ions when taking into account the timeframes for each activity. By enlarging crystal grains, enhancing crystal quality, and passivating flaws, additive engineering is utilized to get rid of or lessen hysteresis [31, 47, 48]. This research reviews the additives used to create stable, effective, and hysteresis-reduced DJ PSCs. We clarify the key characteristics of additives, which are: (i) influencing the morphological characteristics of the DJ perovskite layer; (ii) trying to stabilize the phase of DJ perovskites; (iii) adapting the energy level alignment in DJ PSCs; (iv) repressing non-radiative recombination in DJ perovskites; (v) removing hysteresis; and (vi) boosting the steadiness of DJ PSCs [49]. The workings of the mechanisms and the effects of additions on the functionality of DJ equipment are also evaluated. Despite some encouraging developments, there are still several problems with these DJ-stacked halide perovskites that need basic comprehension and device development.

## Short Outline of the DJ-Layered Halide Perovskites' Structural and Optical Characteristics

The divalent-cation and "large-small" alternating-cation layer configurations [51, 52], which produce significant chemical changes [53, 54], are the essential features that separate DJ from RP phases.

The first difference relates to the crystal formations' octahedral configuration [55]. As shown in Fig. 1, the bulky or long-chain monovalent organic cation pairs in the RP-stacked halide perovskite fill the interlayer gap [32], displacing the neighboring 2D perovskite slabs by half an octahedron [56, 57]. In DJ-layered halide perovskite, however, divalent cations are perpendicularly associated with 2D perovskite pieces [38, 58]. Well-aligned layered structures are produced because there is no shift between octahedral slabs [59, 60]. The focus of the second distinction is the interlayer gap [61], which is clearly affected by the spacer cation properties [62, 63]. Thanks to the bilayer configuration of univalent cation pairs, as indicated in Fig. 1a, interlayer spaces in RP multilayer halide perovskites are frequently the largest [64, 65]. DJ-layered halide perovskite can have significantly lower interlayer distances than RP-layered halide perovskite because their divalent organic cations have a monolayer structure [66–68]. The third difference in layered halide perovskite has to do with how 2D perovskite slabs interact with one another [69]. As seen in Fig. 1a, DJ-layered halide perovskites completely fill the van der Waals gap that appeared in the RP-layered halide perovskite [70]. Through hydrogen bonding, the interlayer cations with double ammonium groupings act together considerably along with the surrounding 2D perovskite pieces [71]. Therefore, the structural stability of 2D-layered halide perovskite may be improved by reducing or removing van der Waals gaps [72]. DJ-stacked halide perovskite has really shown to have extraordinarily good stability [73]. Further hydrogen bond engineering, particularly at high temperatures, can result in the creation of multilayer halide perovskites that are even more stable [74].

We discover that DJ has specific (dis)advantages in relation to the three chemo-structural properties mentioned above when compared to RP-layered halide perovskite [75, 76]. The level of photogenerated carrier dissociation is often greater in DJ-stacked halide perovskites [77, 78] due to the significantly smaller interlayer distances. The stability of DJ multilayer halide perovskite may also be better since hydrogen bonds are present and van der Waals gaps are completely absent [79]. An integrated design of DJ in thin films is therefore essential [50] to achieve the perfect stability between chemical steadiness and carrier transportation, that is widely sought for solar cells. The development of the recorded efficiency for RP and DJ PSCs is seen in Fig. 1b. Table 1 lists all the additives' detailed descriptions for DJ PSC's performances.

**Table 1:** The complete summary of additives for DJ perovskite solar cell performances

Additive	Device structure	Perovskite composition	PCE [%]	Stability	Year [Refs.]
N/A	FTO/PEDOT:PSS/PVK/C60/BCP/Ag	(3AMP)(MA)_3_Pb_4_I_13_	7	N/A	2018 [83]
DMSO	FTO/TiO_2_/PVK/Spiro-OMeTAD/Au	(PDMA)(MA)_3_Pb_4_I_13_	15.8	In air with less than 30% humidity and at room temperature, they perform at over 89% of their original efficiency	2021 [90]
DMSO	FTO/c-TiO_2_/mp-TiO_2_/PVK/Spiro-OMeTAD/Au	( H-BuDA)(Cs_0.20_MA_0.13_FA_0.67_)_4_Pb_5_(I_0.9_Br_0.1_)_16_	10	Under a RH of more than 50% for 1,030 h, they maintained more than 80% of their original efficiency	2022 [94]
DMSO	FTO/TiO_2_/PVK/Spiro-OMeTAD/Au	(PDA)MA_2_Pb_3_I_10_	13.3	Under 40% to 70% RH for 4,000 h with above 95% efficiency	2019 [97]
DMSO	FTO/TiO_2_/PVK/Spiro-OMeTAD/Au	(PDA)MA_3_Pb_4_I_13_	13.8	Under 85 °C with a RH of 50%–70% for 30 days, retains more than 80% of their original efficiency	2021 [63]
DMSO	ITO/PEDOT:PSS/ PVK /PCBM/Ag	(BDA)FA_2_Sn_3_I_10_	6.43	Over 90% of the original efficiency of the unencapsulated device was still present after 1,000 h in a N_2_ environment	2020 [102]
DMSO	FTO/c-TiO_2_/PVK/Spiro-OMeTAD/Au	(DMePDA)FA_3_Pb_4_I_13_	18.86	Maintained 85% and 90% of the initial values after 1,008 h under 85% relative humidity at 85 ℃	2023 [103]
MACl	ITO/PEDOT:PSS/ PVK /C60/BCP/Ag	(PhDMA)MA_4_(Pb_0.5_Sn_0.5_)_5_I_16_	12.2	The stability of DJ phase 2D Pb–Sn PSCs based on PhDMA^+^ is inferior compared with that of 3D Pb–Sn PSCs, resulting from the hydrophilia and poor morphology of (PhDMA)MA_4_(Pb_0.5_Sn_0.5_)_5_I_16_	2022 [105]
MACl	FTO/c-TiO2/mp-TiO_2_/ PVK /Spiro-OMeTAD/Au	(BDA)(Cs_0.1_FA_0.9_)_4_Pb_5_I_16_	18.00	After 100 h of storage at 85 °C, the PCE had lost 20% of its initial value	2020 [106]
MACl	ITO/PEDOT:PSS/ PVK /PCBM/LiF/Al	(BDA)(MA)_4_Pb_5_I_16_	17.91	84% of the original performance is still there after 1,182 h of storage (humidity: 60% RH)	2019 [108]
MACl	ITO/PEDOT:PSS/ PVK /PCBM/LiF/Al	(BDA)MA_4_Pb_5_I_16_	16.38	20 days with 45% RH results in a retention of 80% of original performance	2019 [109]
MACl	ITO/SnO_2_/perovskite/Spiro-OMeTAD/Au	(BDA)(FA_x_MA_1-x_)_4_Pb_5_I_16_	19.55	Maintained 74.89% of the initial PCE after 240 h at 60 °C in an ambient atmosphere (relative humidity 25%–35%), while the control device only remained at 60.57% of original efficiency	2023 [110]
MACl	ITO/PEDOT:PSS/PVK/PCBM/BCP/Ag	(BDA)MA_3_Pb_4_I_13_	12.81	After 23 days in a normal, dark atmosphere (50%–60% RH), 84.3% of the original PCE is still present	2020 [111]
MACl	ITO/PEDOT:PSS/ PVK /C60/BCP/Ag	(PDA)(MA)_3_Pb_4_I_13_	13.0	90% of the original performance is still there after 1,000 h of storage (humidity: 85% RH)	2018 [112]
FACl	ITO/SnO_2_/perovskite/Spiro-OMeTAD/Ag	(PDA_0.9_PA_0.2_)(FA)_3_Pb4I_13_	16.0	After 800 h of aging at 85 °C, 90% of its original efficiency is still there	2022 [107]
MACl	FTO/TiO_2_/ PVK / Spiro-OMeTAD /Au	(DMAPA)MA_(3)_Pb_(4)_I_(13)_	15.16	At 85 °C in air for 1,000 h, more than 90% of the original PCE was still present without encapsulation	2020 [113]
MACl	ITO/PEDOT:PSS/PVK/PCBM/BCP/Au	(PXD)(MA)_2_Pb_3_I_10_	15.6	After 1,500 h in the glove box or 700 h of constant lighting, retains about 90%	2020 [115]
MACl	ITO/SnO_2_/ PVK /Spiro-OMeTAD/Au	(TFBDA)MA_9_Pb_10_I_31_	15.24	After being exposed to ambient air (40%–70% RH) for 1,300 h, retains more than 90% of its original PCE	2021 [116]
NH_4_Cl	ITO/SnO_2_/ PVK /Spiro-OMeTAD/Au	(BDA)FA_4_Pb_5_I_16_	16.75	At the RH range of 15%-20% for 1,600 h, it still retains 93%	2021 [118]
MACl	ITO/PEDOT:PSS/ PVK /PCBM/BC P/Ag	(TTDMA)(MA)_3_Pb_4_I_13_	18.82	After 4,400 h in N_2_, an unencapsulated device may retain, on average 99% of its original efficiency	2021 [85]
MACl	ITO/PEDOT: PSS/PVK/PCBM/BCP/Ag	(ThDMA)(MA)_4_Pb_5_I_16_	15.75	Maintains more than 95% of its initial effectiveness after 1,655 h of storage in N_2_	2020 [101]
NH_4_SCN	ITO/PTAA / PVK /PCBM/BCP/Ag	(BDA)(MA)_4_Pb_5_I_16_	14.53	Retains 85% of their initial PCE under a RH of 50 ± 5% for 900 h	2020 [125]
MASCN	ITO/PTAA/PFN/ PVK /PCBM/BCP/Ag	(3AMP)(MA_0.75_FA_0.25_)_3_Pb_4_I_13_	16.2	After 35 days of storage in air with a RH of 45%, retains 80%	2020 [128]
MASCN	ITO/(NiOx/PTAA)/ PVK /PC61BM/BCP/Ag	(3AMP)(MA_0.75_FA_0.25_)_3_Pb_4_I_13_	18.6	Retains 90% of its original PCE after 60 days of storage in air with a humidity of 45%-50% or 480 h of storage in an environment of 85 °C N_2_	2021 [129]
NH_4_SCN	ITO/c-TiO_2_/SnO_2_/PVK/Spiro-OMeTAD/Au	(NDA)(MA)_(3)_(Pb)_(4)_(I)_(13)_	15.08	Retains 75% after 1,000 h at 60% RH	2022 [37]
NH_4_SCN	FTO/SnO_2_/ PVK /Spiro-OMeTAD/Au	(CHDA)MA_(3)_Pb_(4)_I_(13)_	15.01	Maintains heating efficiency for 68 h at 74.4% at 70 °C and 96.5% at 60 °C	2020 [132]
HI	FTO/PEDOT: PSS/ PVK /C60/BCP/Ag	(3AMP)(MA_0.75_FA_0.25_)_3_Pb_4_I_13_	12.04	Under continuous AM 1.5 G illumination in room air (50%–70% RH) for 48 h, unencapsulated devices retained 22% of their original PCE	2019 [135]
HI	ITO/TiO_2_/ PVK /Spiro-OMeTAD/Au	(4AMP)MA_3_Pb_4_I_13_	16.53	Roughly maintains 90% after 1,000 h	2020 [100]
HI	FTO/PEDOT: PSS/ PVK /C60/BCP/Ag	(3AMPY)(MA)_3_Pb_4_I_13_	9.20	N/A	2019 [137]
HI	FTO/c-TiO_2_/mp-TiO_2_/ PVK /Spiro-OMeTAD/Ag	(BDA)PbI_4_	1.1	N/A	2017 [138]
HI	FTO/TiO_2_/ PVK /Spiro-OMeTAD/Ag	(BDA)PbI_4_	1.08	After 96 h of storage, retains 73.8% of the original performance	2016 [139]
HI	ITO/PEDOT:PSS/ PVK /PC61BM/Bphen/Ag	(PDMA)(MA)_5_Pb_6_I_19_	11	When compared to 3D MAPbI_3_, the PDMA-6 film and device had much better ambient stabilities at 25 °C and 30% RH	2019 [141]
SnF_2_	ITO/PEDOT:PSS/ PVK /PCBM/Ag	(BDA)FA_2_Sn_3_I_10_	6.43	After 1,000 h in a N_2_ atmosphere, an unencapsulated cell retained more than 90% of its original efficiency	2020 [102]
SnF_2_	FTO/TiO_2_/ PVK /PTAA/Au	2D FASnI_3_	7.14%	After aging for more than 1,000 h, the encapsulated gadget still maintained 96% of its original efficiency	2017 [92]
SnF_2_	FTO/TiO_2_/ PVK /PTAA/Au	(PN)_0.1_FA_0.9_SnI_3_	5.85%	N/A	2018 [147]
SnF_2_	ITO/PEDOT:PSS/ PVK /C60/BCP/Ag	(HDA)_0.01_FA_0.99_SnI_3_	7.6	After more than 550 h of storage in the nitrogen atmosphere, retains more than 80% of their original PCE	2020 [148]
SnF_2_	ITO/PEDOT:PSS/ PVK / C60 /BCP/Ag	(PDMA)(FA_0.7_MA_0.3_)_3_(Pb_0.5_Sn_0.5_)_4_I_13_	20.5	About 95% of the original efficiency is still there after 700 h in the N_2_-filled glove box	2021 [150]
SnF_2_	ITO/PEDOT:PSS/ PVK /PCBM/Ag	(3AMP)(MA_0.5_FA_0.5_)_3_(Pb_0.5_Sn_0.5_)_4_I_13_	20.09	After 100 h of exposure to ambient air with 20%–50% RH and sustained AM1.5G light soaking, the PVK cell with 3-AMP preserved over 70%	2020 [153]
FA + doping	FTO/TiO_2_/ PVK /Spiro-OMeTAD/Au	(PDA)[(FA)_0.1_(MA)_0.9_]_3_Pb_4_I_13_	14.74	Sustains more than 92% after 6,000 h of storage at a constant RH of 65% and 800 h of heating at 85 °C in air	2021 [161]
CDTA	ITO/SnO_2_/Perovskite/SpiroOMeTAD/Ag	(BDA)FA_4_Pb_5_I_16_	16.07	Maintains 86% after 360 h of age at 60 °C and 92% after 360 h of aging under one sun's light	2021 [162]
1-methyl-2-pyrrolidinone	ITO/PTAA/ PVK /PCBM/BCP/Ag	(BDA)MA_4_Pb_5_I_16_	16.19	Maintains 83.6% after 700 h of constant 100 mW cm^−2^ illumination	2022 [163]
halogen component regulation	ITO/PEDOT:PSS/ PVK /PCBM/Ag	4AMPY SnX_4_	5.03	No discernible efficiency decline was observed after 200 h in an ambient atmosphere (RH = 30%, T = 25 °C)	2022 [166]
C_3_N QDs	FTO/TiO_2_/ PVK / Spiro-OMeTAD/MoO3/Ag	2D CsPbI_3_	15.63	Due to its favorable phase distribution and enhanced morphological quality, the modified 2D CsPbI_3_ demonstrates long-lasting stability	2022 [120]
BMIMBF_4_	FTO/c-TiO_2_/m-TiO_2_/ PVK /SpiroOMeTAD/Au	(PDA)MA_4_Pb_5_I_16_	14.07	72% of the original value is retained after 120 h of annealing at 85 °C	2022 [173]
BDAI_2_/BAI	ITO/PTAA/ PVK /C60/BCP/Ag	BDAI_2_	18.34	Demonstrating almost 0% efficiency decline over 800 h of continuous thermal aging (60 °C)	2022 [110]
Cs	FTO/SnO_2_/ PVK /Spiro-OMeTAD/Au	(PDA)Cs_x_(MA)_3−x_Pb_4_I_13_	18.30	95% of initial efficiencies were maintained despite 5,000 h of constant one-sun illumination, 240 h of moist heat at 85 °C and 85% relative humidity, and 1,000 h of MPP monitoring	2022 [174]
MAAc	ITO/PTAA/ PVK /C60/BCP/Ag	PeDAMA_3_Pb_4_I_13_	18.41	Maintained 80% of initial efficiency after being kept under 85 °C after 3,000 h	2022 [175]
TU	ITO/PEDOT:PSS/ PVK /PCBM/BCP/Ag	(BDA)(MA)_4_Pb_5_I_16_	12.16	Retained over 95% efficiency upon exposure to ambient air (40% − 70% relative humidity) for 4,000 h	2022 [176]

## DJ-Layered Halide Perovskites Materials and Devices

**Fig. 2:**
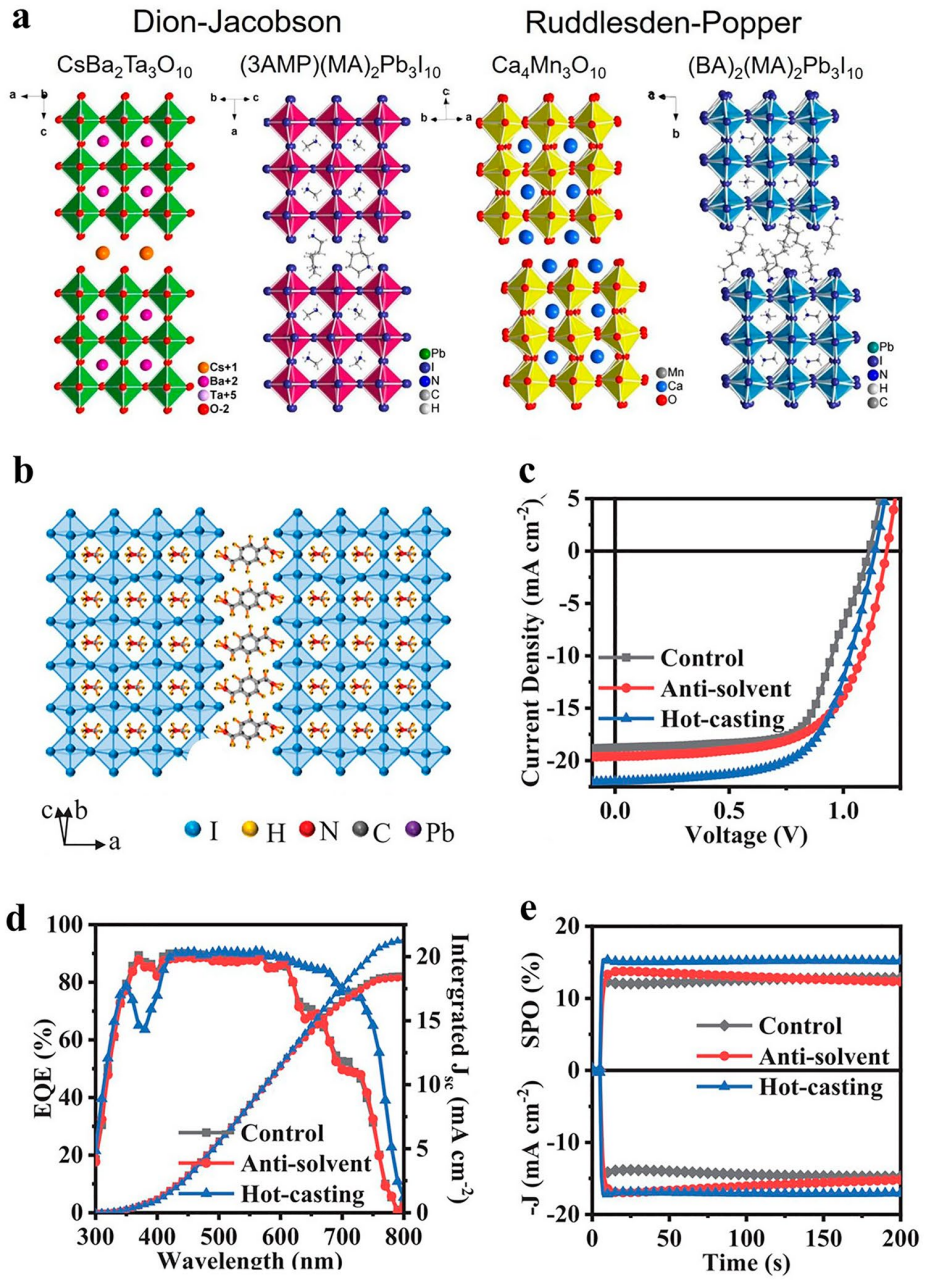
**a** Compare the Dion–Jacobson and Ruddlesden-Popper phases for perovskites made of oxide and halide. Crystal structure of CsBa_2_Ta_3_O_10_, Ca_4_Mn_3_O_10_ and (BA)_2_(MA)_2_Pb_3_I_10_ [83]; Copyright 2018, ACS. **b** An illustration of the crystal architecture in a schematic of the 2D-DJ perovskite (PDMA)(MA)_n−1_Pb_n_I_3n+1_ (n = 4); **c** the hot-casting, antisolvent, and control devices' J-V curves and solar cell design, respectively; **d** For the three devices' champion cells, EQE and integrated short circuit current density (*J*_sc_); **e** The steady-state power (SPO) and current density for the three devices were tested for 200 s at a permanent Voc close to the MPP [90]. Copyright 2021, Wiley–VCH

Nonradiative recombination severely reduces the efficiency of solar cells. Defects are inevitable since perovskite films are often polycrystalline [17, 80]. The most common cause of nonradiative recombination is a carrier recombination center caused by a defect [51, 81]. There are many different types of flaws in solution-processed DJ halide perovskite films, including dislocations, grain boundaries, and point flaws like vacancy [82], interstitial, and antisite substitution defects [50]. Although research has shown that the majority of these defects are light carriers' traps and do not cause significant nonradiative recombination [79], cautious modification of defects in DJ halide perovskites is still necessary to advance DJ PSC performance toward the Shockley-Queisser efficiency limit [78]. In Fig. 2a, a novel crystal pattern established on the DJ class of PVK creates 2D hybrid PVK devoid of additives, as examined by the Mao research team [83]. These materials are composed of layers, and the precise arrangement of the inorganic layers one on upper of the other gives the filling of these layers a distinctive appearance. The use of the different spacer cations (3AMP and 4AMP) significantly affects the general properties. The 3AMP and 4AMP exhibit lesser band gaps than the most widely used 2D-RP perovskites as a result of a fewer distorted inorganic structure and nearer interlayer gaps. The actual device assembly demonstrated that the 3AMP series had superior optoelectronic properties over the 4AMP series. The winning gadget, according to the preliminary findings, has a PCE of more than 7%. It has been revealed that nonradiative recombination-causing flaws in 2D-DJ-layered halide PVK layers are typically present at grain boundaries or at the interfaces of PVK and choosy connections [76]. The bulk of these faults are uncoordinated ions like I^−^ or Pb^2+^ [84] and faults are divided into two types: positive defects and negative defects. This is due to the periodicity of the termination lattice. As a result, a variety of compounds might be used to passivate flaws [31, 85].

### *N*, *N*-dimethyl Sulfoxide Additives

Different types of additives are used in the manufacture of DJ PSCs [86]. The strong coordination capability of Pb^2+^ and I^−^ in DJ halide PVK [87], that additionally serves as the basis for PSC solution procedure [88, 89], is primarily responsible for the diversity of accessible additives. Ions from salt additions can coordinate with the lead cation or iodine anion from DJ perovskites thanks to an ionic connection, which is well understood. Via exhausting an antisolvent rinsing stage throughout spin-coating of the perovskite solution, Zhang et al. [90] proved a solvent manufacturing technique. This caused fast supersaturation for even, dense nucleation, creation of perovskite grains, which resulted in the development of a dense, flat, fully enclosing, extremely crystalline DJ perovskite thin layer in Fig. 2b-e. The Lewis base *N*, *N*-dimethyl sulfoxide (DMSO) was utilized to create a stable and homogeneous intermediate phase of MAI, PbI_2_, and DMSO, which was then used to anneal into perovskite to slow down the interaction between MAI and PbI_2_. As a result, carrier movement along the perpendicular direction was considerably boosted and power conversion efficiency was increased. The efficiency of the hot-casting equipment was excellent at 15.81%.

In order to generate a Lewis adduct and passivate the defects, Lewis bases often serve as electron donors that are able to attach to the clearly charged, undercoordinated Pb^2+^ [83, 91–93]. According to Cohen et al. [94], PbI_2_∙DMSO adducts are created when Lewis bases DMSO and/or iodide interact with Lewis’s acids PbI_2_, and it has been demonstrated that these adducts are essential on behalf of advanced charge extraction and a gentler rate of recombination in the H_3_N–C_4_H_6_(OH)_2_–NH_3_ (*n* = 5) spacer-based low-dimensional DJ perovskite. Accordingly, the DJ solar cell demonstrated improved stability and a 10% efficiency during 1030 h of relative humidity (RH) of more than 50%. A Lewis base is an oxygen-containing lone pair of electrons which coordinately bonds to undercoordinated PbI_2_ [95]. It has been discovered that several solvents, such as DMSO, are polar aprotic and include an oxygen-bearing lone electron pair, indicating that they can function as O-donor Lewis bases [96]. By replacing DMSO with -butyrolactone (GBL), Ahmad et al. [97] showed that a steady intermediate adduct phase is able to form. This phase can then be converted into homogeneous and fresnel lens Dion–Jacobson phase 2D (PDA)MA_2_Pb_3_I_10_ perovskites, which provide PCE of 13.3% as well as extremely high device stability.

DMSO can provide intermediates that can make crystallized and great DJ perovskite layers [6]. Applying a hybrid solvent of GBL and DMSO, Fu et al. [63] evolved solvent manufacturing to generate a consistent, good DJ (PDA)MA_3_Pb_4_I_13_ (*n* = 4, PDA stands for 1,3-propanediammonium) PVK layer for high-performance PSCs by a credentialed PCE of 13.8% and brilliant thermal steadiness at 85 °C in humid atmosphere. PbI_2_ solubility was increased when DMSO was used, and the crystallization kinetics were adjusted for significantly better film coverage and smoothness. A larger monoammonium is added to the diammonium-based 2D-DJ-DMSO perovskite as the matrix to produce a second RP phase PVK that coexists with DJ PVK. This helps with crystal formation, reduces charge recombination, and enhances charge transport.

DMSO, a very polar solvent that can dissolve lead halide, was generally used to dissolve DJ halide perovskites [98, 99]. It has also been shown that DMSO can be used to make excellent DJ perovskite film based on tin (Sn) [100–102]. Li et al. [102] used solvent-controlled perovskite film development to achieve a record PCE of 6.43% based on Sn-based DJ (BDA)FA_2_Sn_3_I_10_. The reaction between DMSO molecules and Lewis' acid Tin (II) iodide (SnI_2_) produced the intermediate adduct SnI_2_·3DMSO, that competently slows the reaction between SnI_2_ and methylammonium iodide (MAI), producing an even and pinhole-free MASnI_3_ layer by micron-sized grains afterward the DMSO molecules are removed by annealing at 100 °C. The intermediate's crystal structure was determined to be SnI_2_·3DMSO, with the coordination sphere being made up of three DMSO molecules that are connected to Sn by oxygen and iodide ions. Additionally, (BDA)FA_2_Sn_3_I_10_ displayed strong crystal symmetry, which led to efficient carrier separation and absorption. The compact (BDA)FA_2_Sn_3_I_10_ film displayed weaker quantum confinement with better carrier diffusion and mobility, according to transient absorption (TA) studies. However, the (BDA)FA_2_Sn_3_I_10_ devices demonstrated greater resistance to oxidation, light, and humidity.

**Fig. 3:**
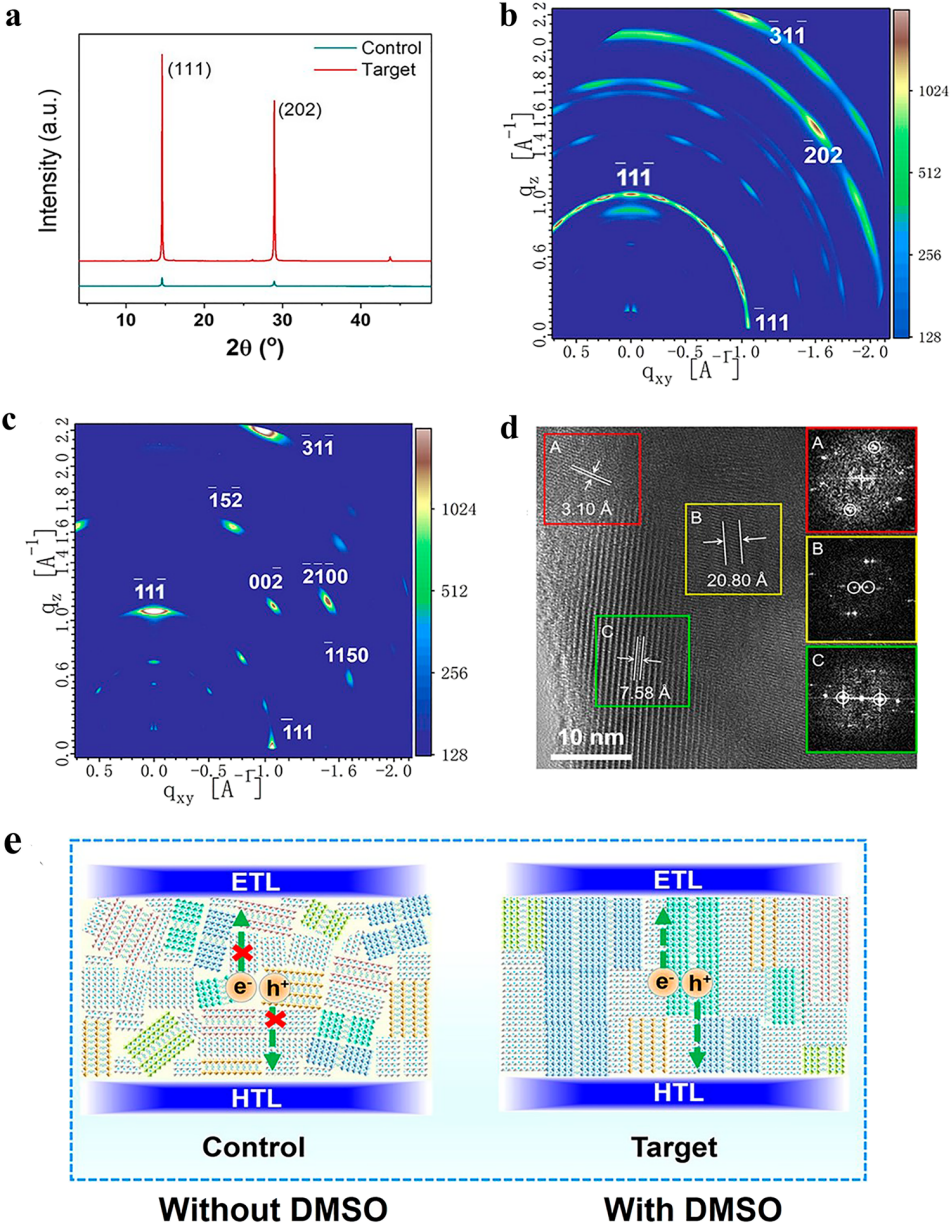
**a** The control and target layers' XRD patterns; **b, c** GIWAXS data for the reference and target movies; **d** Target film's HRTEM picture. FFT pictures of the relevant region are shown in the insets; **e** Diagrammatic representation of the control and target cells' morphologies and charge-transport schemes [101]. Copyright 2020, ACS

The performance of the device was improved when DMSO additive was added to the (ThDMA)(MA)_*n*-1_Pb_*n*_I_3*n*+1_ (*n* = 5) precursor solution, along with Lu and coworkers [101] (Fig. 3). A process utilizing a combined solvent of DMF and DMSO might be used to create significant 2D-DJ perovskite, as well as better crystallinity, desired vertical orientation, and expanded spatially determined carrier life. The enhanced device demonstrated a good PCE of 15.75%, that was a record, for aliphatic spacer-based 2D-DJ PSCs. The unconjugated 2D-DJ perovskite cells also retained around 95% after 1,655 h in N_2_. These findings imply that the design of thiophene-based aromatic spacers and the engineering of devices may result in extremely effective and reliable 2D-DJ PSCs.

Xiang’s study [103] recognized a facile green-antisolvent procedure with DMSO additive to control the phase delivery of MA-free quasi-2D DJ phase PVK films (DMePDA)FA_3_Pb_4_I_13_ (*n* = 4). The layers offered a more homogeneous phase delivery, resulting in higher out-plane mobility, decreased surface-trap density, and better conductivity. The PCE of 2D-DJ-layered halide PSCs and the shape of perovskites were both shown to be significantly enhanced by DMSO-induced complexes.

### Chlorine Anion Additive

Chloride is a common addition for passivation (Cl^−^) [85]. Cl^−^ significantly lowers trap density and enhances charge durations of diffusion and carrier lives in DJ halide perovskite films [104, 105]. DJ phase Pb–Sn PVK of (PhDMA)MA_4_(Pb_0.5_Sn_0.5_)_5_I_16_ in 2D PSCs is used with MACl additive [105]. MACl is adopted to enhance the layer quality of 2D Pb–Sn PVK, enabling the suppression of the trap density and nonradiative recombination for DJ PSCs. Zhao’s research team [106] recently developed a technique for solvent vapor plasticizing 2D PVK layers by addition of 20 mol% methylammonium chloride (MACl) additives. Their rotation caused by the surface and accompanying grain development were assisted by MACl, which improved out-of-plane charge transfer. Comparing 2D perovskite solar cells to their untreated counterparts, they are more efficient and stable. Their (BDA)(Cs_0.1_FA_0.9_)_4_Pb_5_I_16_ solar cell showed a certified PCE of 18.00%.

Cl^−^ is crucial for effective planar DJ PSCs in the early stage [107] because it passivates flaws in DJ perovskite films and on the surface of titanium dioxide's (TiO_2_) ETL and at the interface between TiO_2_ and DJ perovskite. Significantly, (BDA)-based PSCs were created by Niu and colleagues [108]. To help the perovskite (PVK) film crystallize and produce big, high-quality PVK grains, they applied the MACl additive. The BDA caused the inorganic sheet gap to be smaller and the inorganic sheets to be more ordered, which allowed the PVK layers to present favorable charge movement, very few defects, and almost crystallinity with a single crystal. The PCE of the solar cells that performed the best was 17.91%, and there was negligible hysteresis. The PSCs demonstrated high stability as well; after 1,182 h of storage (humidity: 60% RH) in room air without any encapsulation, 84% of the original PCE was still present. Even though the majority of these research primarily focused on morphology and demonstrated that the chloride residual in the film A rising (BDA)MA_4_Pb_5_I_16_ film was produced by Zheng et al. [109] using MACl in precursor solution. The formation energies demonstrated that the perovskite construction is thermodynamically stabilized by Cl^−^. Because to the excellent surface shape, crystallographic characteristics, and optical absorption properties, the device displayed an appealing PCE of 16.38%.

Common processes like the antisolvent approach and sequential deposition technique for producing DJ perovskite films often include chloride additives. Jin [110] have studied a novel method with MACl additive to better remove the surface traps in PVK layers by the additional secondary anti-solvent treatment. A highest PCE of 19.55% is completed based on DJ PVK. Recently, Wang et al. [111] reported that the delivery uniformity is significantly enhanced with a temperature fabrication method by adding Cl^−^ to the MAI precursor solution and using (BDA) as cross-linking molecules to generate 2D-DJ PSCs. By using MACl and/or DMSO as additives, the dispersity and crystalline orientation may be adjusted, leading to the production of perovskite films of excellent quality. It produced (BDA)MA_3_Pb_4_I_13_ recipes with PCEs up to 12.81% efficiency. MACl is one of the additives utilized most often to create superior PVK layers among these organic ammonium salts [39]. Ma et al. [112] created a (PDA)-based 2D perovskite cell and used MACl additive, which was found all over the film and was integrated into the perovskite lattice, to obtain a PCE of 13.0%. The increased connection across the perovskite structure also contributes to the steadiness of the PDA-based DJ PVK. The findings show that 2D perovskites with low interlayer distances function very well, offering an alternate method for enhancing the effectiveness and stability of 2D perovskites. They concluded that the secret to good performance is Cl^−^.

**Fig. 4:**
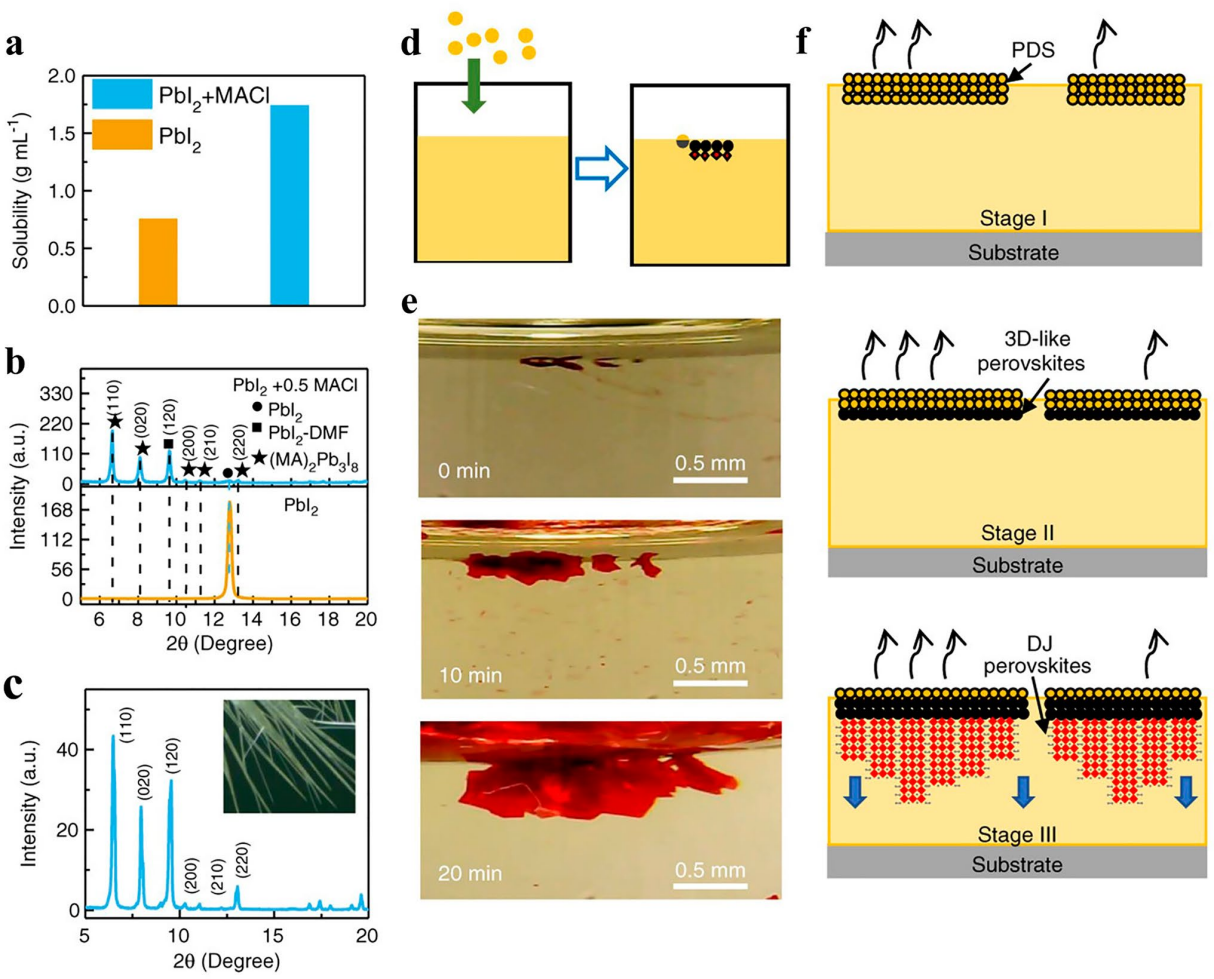
**a** PbI_2_'s solubility in DMF both with and without 0.5 MACl; **b** XRD patterns of the layers made from raw PbI_2_ and PbI_2_ with 0.5 MACl; **c** Optical picture of the intermediate phase fiber made from (PXD)(MA)_2_Pb_3_I_10_ and its associated XRD pattern; **d–e** Graphics and visual images of the directed formation of PXD DJ PVK crystals at the liquid surface of the oversaturated precursor solution (n = 2) under the purposefully added (PXD)(MA)_2_Pb_3_I_10_ powder. **f** The right panel shows how the directed development of DJ perovskites is caused by the production of 3D-like perovskites on a PbI_2_-N,N-dimethylformamide (DMF)-based solvated phase (PDS) surface that has been soaked in DMF [115]. Copyright 2020, Wiley–VCH

Additionally, a secondary interlayer spacer, Lead (II) chloride (PbCl_2_), has been added to low-dimensional perovskites based on formamidinium (FA), which significantly enhances the film quality [90, 107]. According to Zhao and colleagues [113], the Cl additive can greatly lengthen the diffusion lengths of the hole and electron. Additionally, it lowers the PVK thin film's bulk trap-state density [114]. Recent research by Wang et al. [115] focused on the nucleation of DJ (PXD)(MA)_2_Pb_3_I_10_ perovskite layers with the MACl additive. For DJ perovskites with large organic cations, this is accurate, such as p-xylylenediamine (PXD) in Fig. 4. Due to its low sublimation temperature, the majority of MACl was eliminated during the thermal annealing. Pure perovskite films were produced, improving coverage and absorption, which boosted device performance. For PSCs based on (PXD)(MA)_2_Pb_3_I_10_, the engineering resulted in a dramatic improvement in PCE, going from 1.2% to 15.6%. Even after 1,500 h of storage in a glove box or 700 h of continuous light, the unencapsulated DJ perovskite devices still had an efficiency of above 90%.

**Fig. 5:**
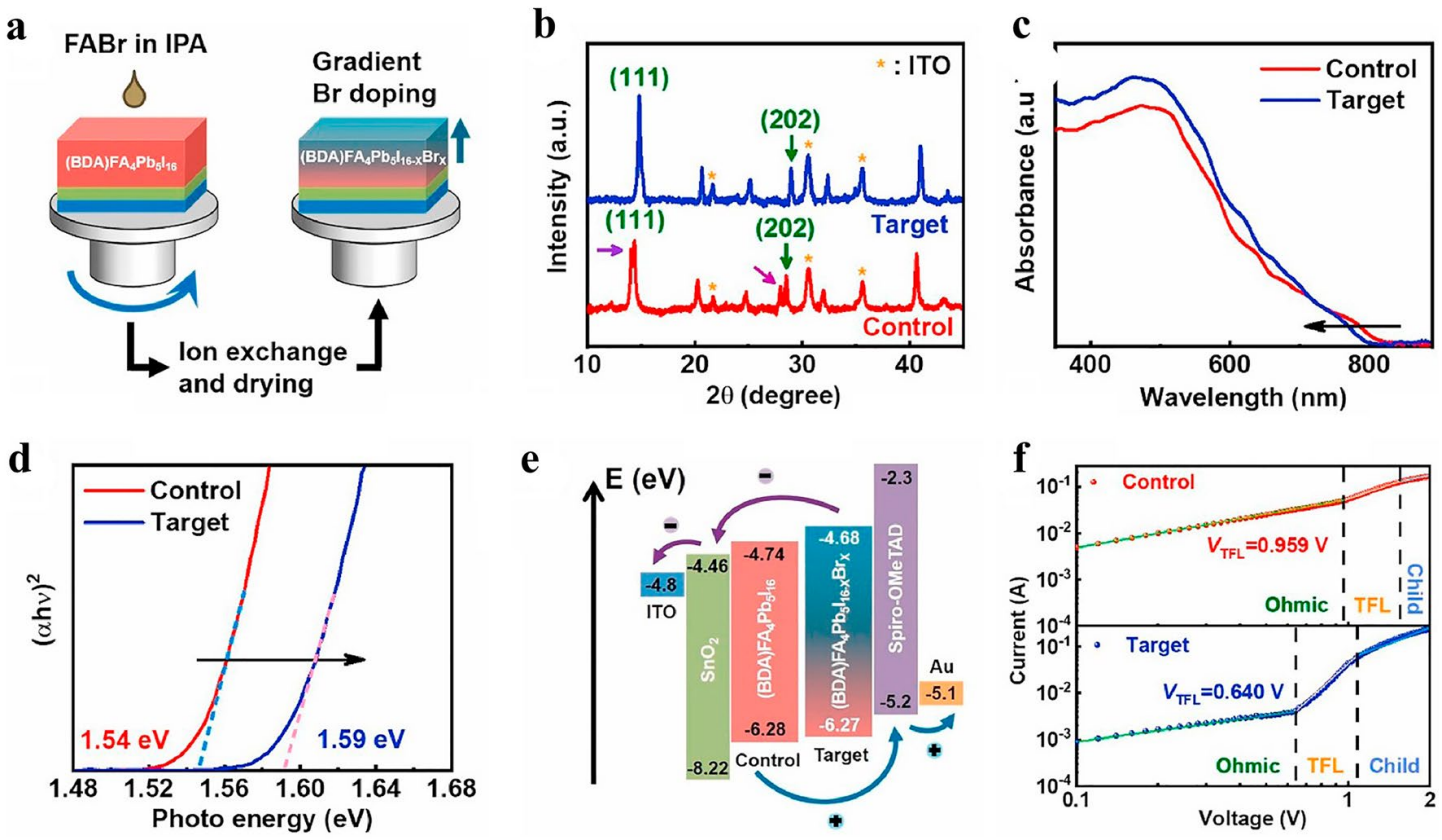
**a** Diagram of the preparation procedure in a schematic of (BDA)FA_4_Pb_5_I_16-x_Br_x_; **b** Perovskite films' XRD patterns both with and without FABr post-treatment; **c** Spectra of UV–vis absorption and **d** the perovskite layers' band gap both before and after FABr treatment; **e** Diagram of the PSC's energy levels dependent on the control and desired PVK layers; **f** Dark I-V curves. [118] Copyright 2021, Elsevier

Wang et al. [116] also discovered that 2,3,5,6-tetrafluoro-1,4-benzenedimethanammonium (TFBDA^+^) may be utilized to create a unique DJ PVK layer with dense and evenly dispersed geomorphology and enhanced charge transfer. Cl^−^ slowed down the manufacture of PVK layers and made the PVK layers' color darker. The delayed crystallization procedure raised the film coverage on the planar substratum and considerably enhanced the device presentation of the mesoporous cell architectures. The unconjugated cells reserved more than 90% of their fresh efficiency after 1,300 h in 40%-70% RH air more than 80% that after 100 h of annealing process at 80 °C, demonstrating its noticeably enhanced water as the base and thermal performances compared to its nonfluorinated counterpart. Larger-sized cations as MA^+^, FA^+^, or (ammonium) NH_4_^+^ are used as additions in chloride salts [117]. Because of the organic cation's mass, there is a lot of interionic space, which reduces the strength of the electrostatic attraction that attracts electrons to one another [47]. In the PVK annealing post-treatment, the organic chloride addition may be simply removed because chloride is combustible [89]. Additionally, researchers have investigated how NH_4_Cl additive affects the crystallinity and surface morphology of perovskites. Su [118] et al. showed that NH_4_Cl improves crystallinity and morphology of (BDA)FA_4_Pb_5_I_16_ perovskite thin films in Fig. 5. Beginning with the gradient energy band alignment, carrier transit, extraction, and transfer are made simpler. Better crystallinity and a decrease in defect density as a result of recrystallization are seen after FABr treatment. Last but not least, Br inclusion primarily increases device stability. The device's PCE of 16.75%, which was much greater than the control devices of 0.5%, was primarily caused by a massive increase in *V*_OC_ of 1.107 V.

In addition, Xu et al. [85] investigated TTDMAI, a spacer made of fused thiophene that has been effectively produced for 2D-DJ PSCs with Cl^−^. In comparison with the mesoporous design, the planar architecture was shown to have bigger grain sizes and higher interfacial charge injection rates due to chloride doping. The effectiveness of PSCs was greatly enhanced. Cl^−^ was discovered to help crystals form; perovskite films' absorbance or photoluminescence properties were unaffected. It's significant to note that the TTDMA-based unencapsulated device kept its average efficiency at 99% of its starting value despite being stored in N_2_ for 4,400 h. Additionally, compared to their 3D counterparts, light, thermal, environmental, and operational stabilities were all greatly increased.

Cl^−^ ions have been shown to regulate DJ PVK growth and nucleation without interfering with the lattice [119–121]. Due to its volatile nature, it prefers to remain near the grain borders after post-annealing. The crystals are more homogeneous, and the crystal growth rate is lowered [122]. The consequence is larger grains and improved pinhole density in the film morphology. Finally, it improves the DJ cell's photovoltage capability.

### Thiocyanate Anion Additive

PSC performance is greatly influenced by the surface abrasiveness, grain size, and homogenous coverage of the DJ perovskite absorber layer [41, 123, 124]. Although they have a high defect tolerance, organohalide lead perovskites are not defect-proof.

**Fig. 6:**
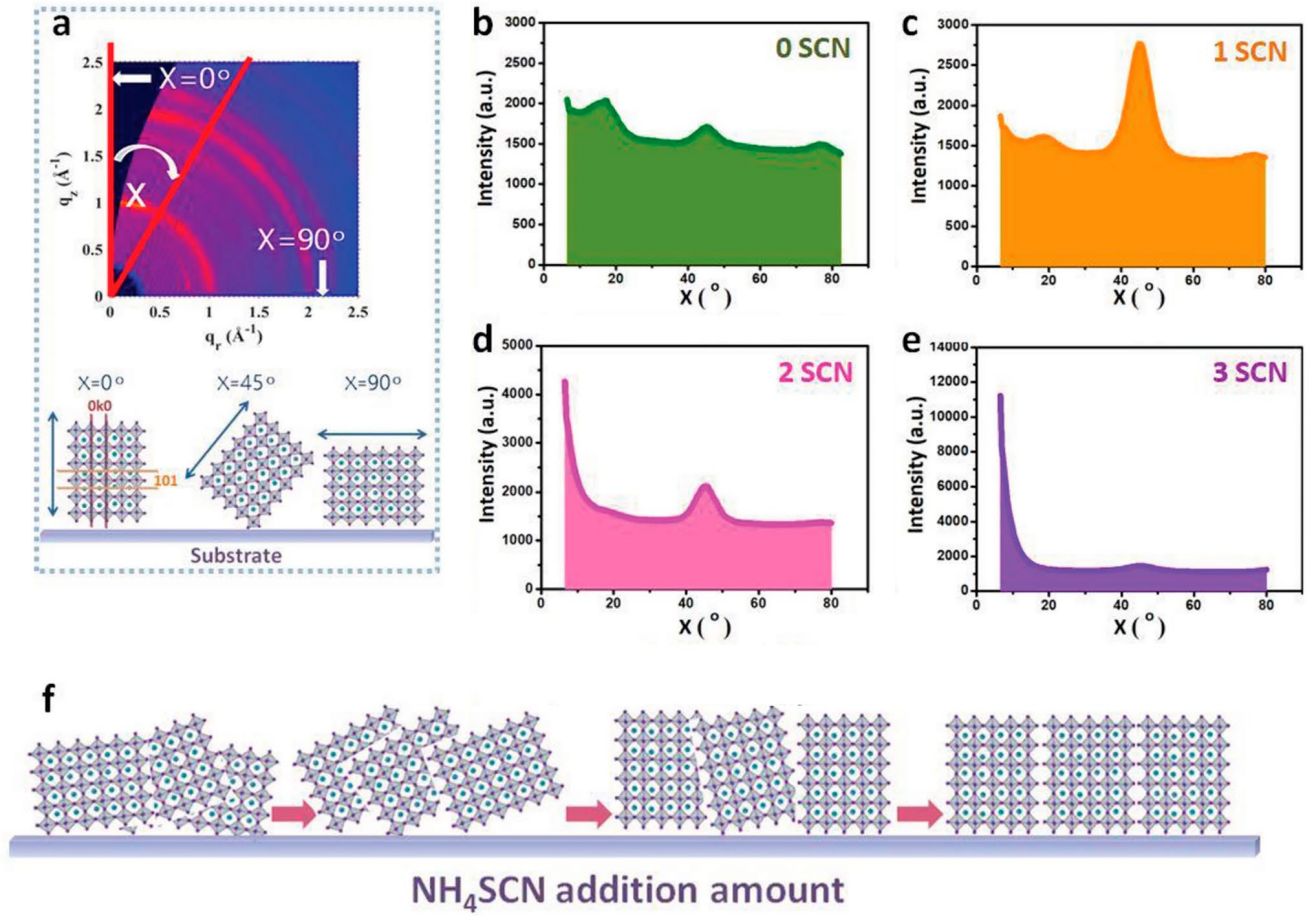
**a** Diagram depicting the progression of the (101) crystallographic plane's azimuth angle; **b–e** Polar intensity profiles along the ring at q = 0.95–1.08 Å − 1 assigned to the (101) plane of PVK layers with various SCN^−^ additives; **f** Diagram showing the development of the crystallographic plane (101)'s orientation as the quantity of NH_4_SCN added increases [125]. Copyright 2020, Wiley–VCH

Li et al. [125] showed that recrystallization caused by ammonium thiocyanate (NH_4_SCN) may be used to enhance the shape and crystalline quality of PVK films (Fig. 6). The additive was largely dissolved when they created the (BDA)(MA)_4_Pb_5_I_16_ solution with NH_4_SCN, and the PbI_2_ compound then formed the mesoporous framework (intermediate film). The NH_3_ and HSCN that were created during the annealing process soon volatilized. Because of the large crystal grain sizes and reduced trap density, (BDA)(MA)_4_Pb_5_I_16_ produced by this intermediate catalytic action of NH_4_SCN had a higher PCE of 14.53%. The devices that were created also had good stability, retaining 85% under RH 50% ± 5% for 900 h. NH_4_SCN and MASCN have been used to enhance DJ PVK films' crystallinity and boost cell performance [3, 126]. The DJ PVK layer's grain size, material crystallinity, and optical properties may be improved by pseudo-halide [103, 127]. This manuscript examined a number of BDA-based 2D DJ perovskites. However, stability of some BDA-based 2D DJ perovskite devices was rather low in comparison with their counterparts of other types. Table 1 demonstrates that testing of 2D DJ PVK solar cells typically takes place in low humidity environments, N_2_ atmospheres, or in the absence of light.

2D (3-AMP) (MA_0.75_FA_0.25_)_3_Pb_4_I_13_ DJ perovskite layers were produced by Wu et al. [128] using a room temperature method and the MASCN additive. With the right number of additives, the perovskite layer displayed improved crystallinity, enhanced orientation growing, and fewer flaws. Planar-structured PSCs had the highest PCE of 16.25%. After 35 days of air storage at a RH of 45%, the unsealed cells still contained 80% of its initial efficiency.

A number of thiocyanate ions have been suggested as ways to regulate the crystal border (GB), charge carrier trapping, and defect tolerance in perovskite thin films. Wu et al. [129] produced (3AMP) (MA_0.75_FA_0.25_)_3_Pb_4_I_13_ films of excellent quality by adding MASCN to the precursor. When the centration is high enough, it is probable that SCN^−^ outcompetes DMSO for coordination sites, reducing the interaction between Pb^2+^ ions and DMSO and facilitating easier DMSO elimination. Reduced fault density allowed for an exceptionally low energy loss and high *V*_OC_ (1.24 V) (0.35 eV). A large PCE of 18.67% was present in a gadget. After storage in the open air, it retained 90% of its original PCE over 50 cycles of thermal cycling testing.

**Fig. 7:**
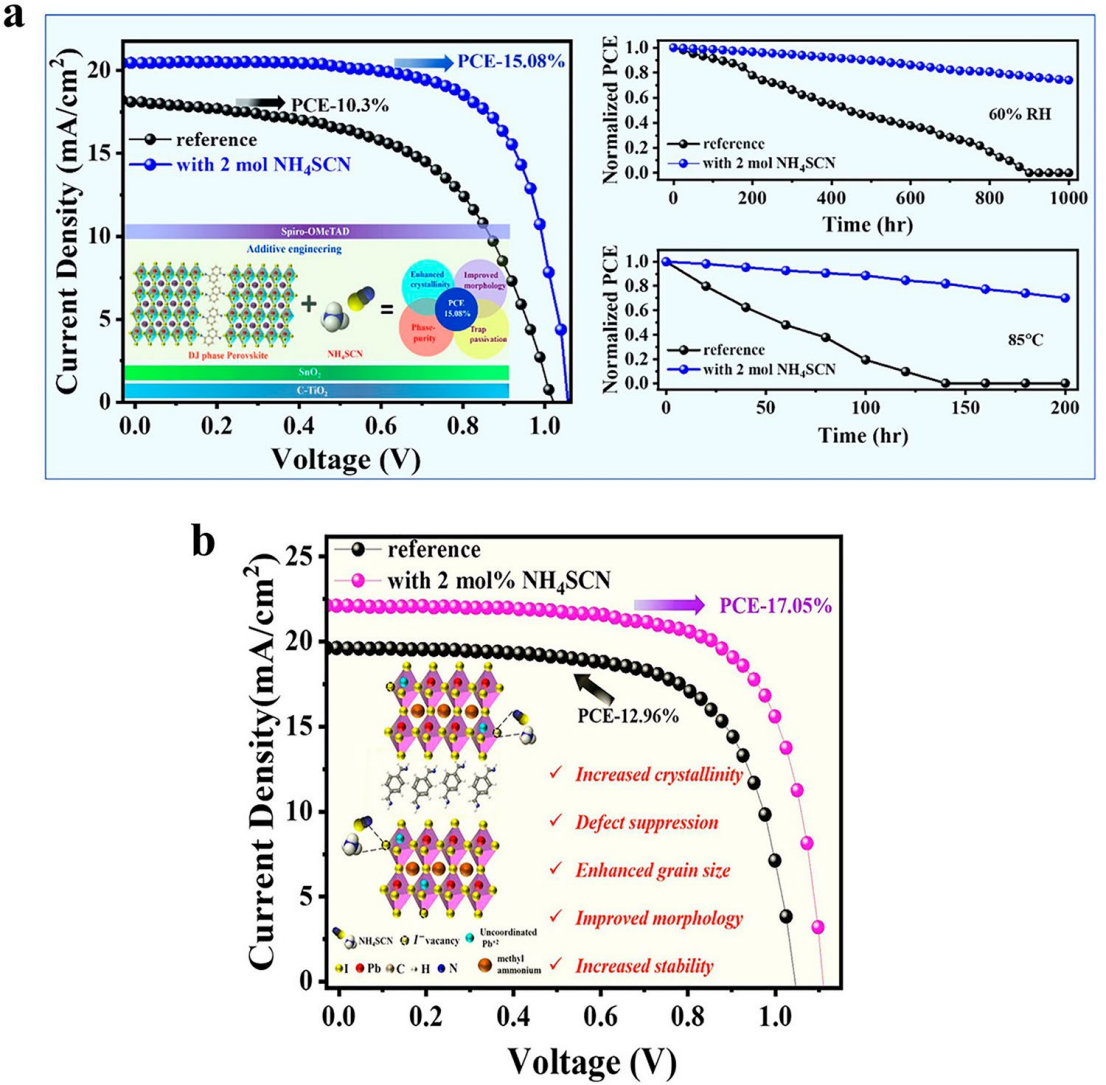
**a** Curves of the control and target cell's current density and voltage; continuous testing of the goal and control devices [37]. Copyright 2022, ACS; **b** Curves illustrating the control and target device's current density vs voltage [131]. Copyright 2022, ACS

Halogen shares similar chemical characteristics and behaviors with pseudo-halides like SCN^−^. Yukta et al. [37] reported the eclipsed DJ perovskite employing the 1,5-diaminonaphthalene cation in Fig. 7a. In NDA-based DJ phase perovskite (NDA is 1,5-diaminonaphthalene), NH_4_SCN surface treatment increased carrier mobility, film shape, crystallinity, trap-assisted nonradiative recombination, and trap-assisted nonradiative recombination significantly decreased. Consequently, the efficiency of perovskite device treated with NH_4_SCN increased by over 46%, from 10.3% to 15.08%. The effectiveness of the devices treated with the NH_4_SCN addition was verified using electrochemical impedance spectroscopy [130, 131]. To date, many pseudo-halides have been incorporated onto DJ perovskite layer in order to change the processes for PVK crystal formation and passivate crystal defects, improving PSC performance.

Trans-1,4-cyclohexanediamine (CHDA)MA_(3)_Pb_(4)_I_(13)_(n = 4) was manufactured via Wang et al. [132] using NH_4_SCN as an addition. They showed that both cation and anion had synergistic effects on the perovskite layer's nucleation and growth, resulting in bigger grains and more stable layers. Less flaws were created, and the crystallization process took longer. The device generated astounding efficiency of up to 15.01%. The encased devices' efficiency remained at 80.7% over 270 min of continuous maximum power point monitoring. Devices manufactured maintained 96.5% and 74.4% efficiency during 68 h at 60 and 70 °C, respectively. One of the maximum stabilized efficiencies for a DJ phase perovskite has been informed by Yukta et al. [131] for (XDA)(MA)_3_(Pb)_4_(I)_13_ PVK **(**Fig. 7b**)**. The ideal phase distribution, bigger grains, and better crystallinity are produced by the NH_4_SCN additive alteration. The passivated perovskite also demonstrated improved charge transport and decreased defect density.

The DJ PVK film has been passivated using the SCN^−^ as an additive [133]. Inorganic perovskite Pb^2+^ may coordinate with the S and N atom donors found in thiocyanate to generate the Lewis acid–base adduct [93]. The SCN ions would cause the perovskite layer to emit gases in the form of methylamine (CH_3_NH_2_) and HSCN through the annealing procedure [57]. The emission of CH_3_NH_2_ gas during perovskite synthesis enhances the crystallinity and grain size of the layers [134].

### Hydrohalic Acids Additive

The perovskite precursor solution is made up of colloids with an organic and inorganic lead polyhalide framework between them [60]. These colloids, that also behave as nucleation sites, control the coverage and form of the thin films that undergo deposition [34]. Large colloids cause poor film morphology [23]. Large colloid particles can be broken down by hydrohalic acids, and they can also change the colloidal concentration [38], which modifies DJ perovskite shape while it is being deposited. Ke et al. [135] discovered that adding a little quantity of HI to a precursor solution allowed for the structure of phase-pure, uniform, and continuous 3AMP (MA_0.75_FA_0.25_)_3_Pb_4_I_13_ layers. Particularly, 3AMP perovskites showed lower recombination, boosting the devices' FF and VOC. The ideal level of 25% FA was present in these perovskites. The solar cells therefore showed a 50% improvement over the original cells, producing a winner efficiency of 12.04% with a great FF of 81.04% and an efficiency of 9.83%. When the solvent-engineering technique is paired with hybrid DMF/DMSO solvent and HI additive, the films have a noticeably more desirable perpendicular orientation and greater crystalline quality, resulting in solar cells with outstanding repeatability and good presentation. Due to the hydrophobic nature of the organic spacer layer of the 3AMP cation, which increases the materials' resistance to moisture and light, these DJ PVK layer cells also demonstrated substantially greater environmental stability than their 2D-RP and 3D analogs.

**Fig. 8:**
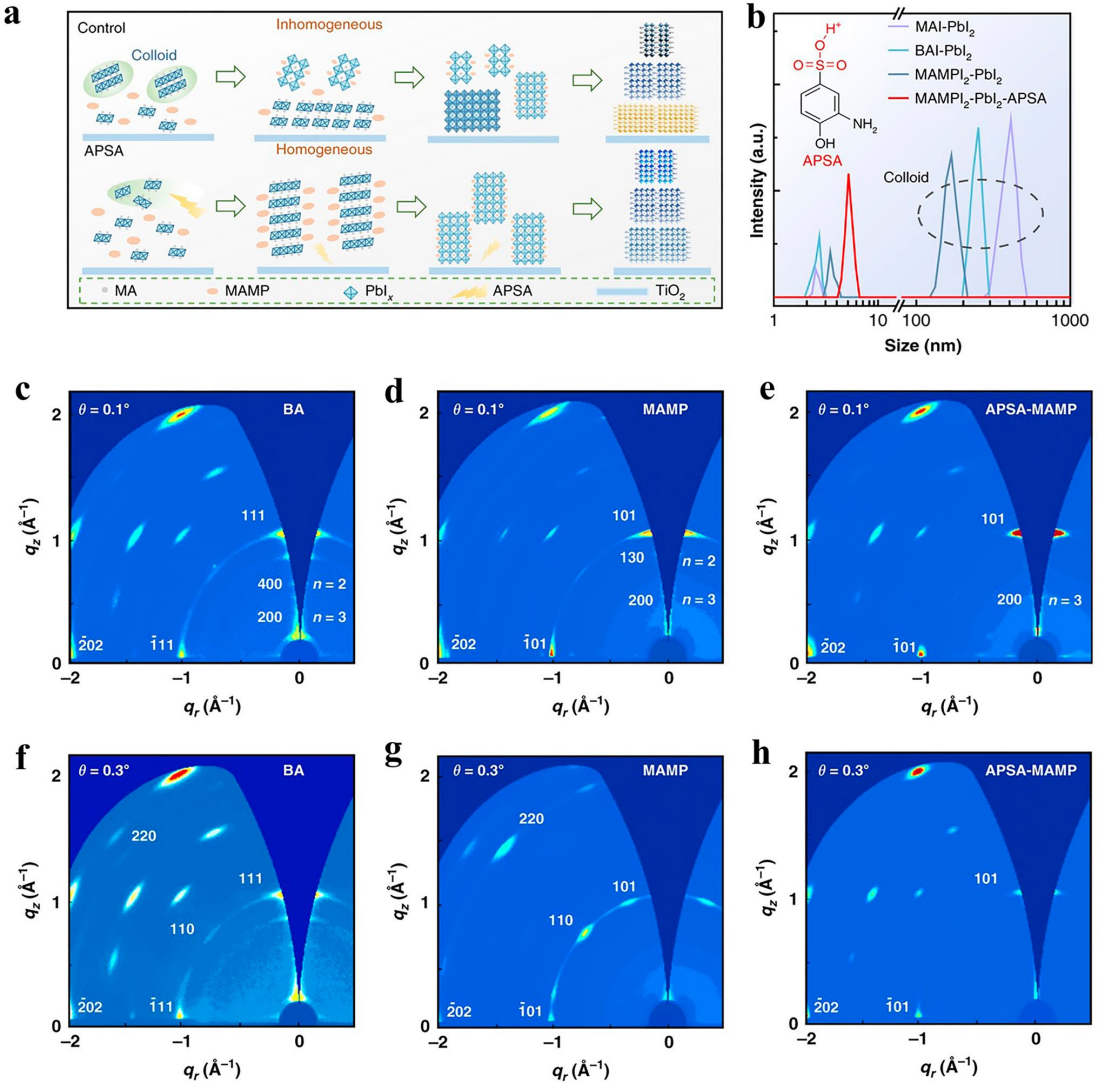
**a** Schematic representations of the crystallization of control and APSA-treated quasi-2D perovskite layers; **b** To assess the colloid size and dispersion in precursor solutions, use dynamic light scattering data. Inset: APSA's molecular construction. Characterizations of GIWAXS for (BA)_2_(MA)_3_Pb_4_I_13_, pristine, and APSA-treated (4-AMP)MA_n−1_Pb_n_I_3n+1_ perovskite films, **c–e** an angle for grazing incidents of 0.1° and **f–h** at an angle of grazing incidence of 0.3°, respectively [100]. Copyright 2020, Nature Publishing Group

The presentation of DJ PSCs as a device and the layer quality of DJ perovskites are both impacted by the aging of DJ perovskite precursors [136]. Controlling the colloid dispersion in the precursor is necessary to produce uniform films [52]. HI was used most frequently as an additive in the early stages of DJ PSC development. He et al. [100] fabricated dense (4-AMP)MA_*n*−1_Pb_*n*_I_3*n*+1_ perovskite films by introducing HI to the (4-AMP)MA_*n*−1_Pb_*n*_I_3*n*+1_ precursor in Fig. 8** (**MAMP is 4-AMP in Figure**)**. The addition of HI could make the (4-AMP)MA_*n*−1_Pb_*n*_I_3*n*+1_ solution more soluble, increasing the surface coverage on the substrate. Furthermore, by meticulously controlling the crystallization kinetics using HI additive, 4-AMP film with a homogenous energy landscape was achieved. Electrical analyses proved that the homogenous energy landscape decreased defect density and lowered energy disorder in the film. Due to the homogenous energy environment, which reduces nonradiative recombination and energy disorder, little V loss was obtained. The solar cell had an impressive V_OC_ of 1.21 V and a noteworthy efficiency of 16.53%. Due to its excellent vertical phase alignment, the device also has shown far more stability than normal hot-casting films, keeping around 90% of the fresh PCE after 1000 h of storage.

In contrast to neutral additives, the addition of acids may change how the molecules of the solvent interact and decrease the viscosity of the solution [11]. This alters the solvent's rate of evaporation and influences how quickly DJ PVK crystallizes. Li et al. developed exceptionally smooth and totally covering PVK layers with big crystalline grains at ambient temperature in this study [137]. The devices had a PCE of 9.20% (*A*'*A*_(*n*-1)_Pb_(*n*)_I_(3*n*+1)_ (*A*' = 4-(aminomethyl)pyridinium (4AMPY), A = methylammonium (MA), *n* = 1–4)).

Acids can make DJ PVK precursors more soluble and aid in the production of DJ PVK films that are uniform and well-covered. Subsequently, Safdari et al. [138] used HI into [NH_3_(CH_2_)_4_NH_3_]PbI_4_ and successfully prepared stable cubic [NH_3_(CH_2_)_4_NH_3_]PbI_4_ films. It was shown that the stable black phase could only be created at low temperatures due to the smaller grains and lattice strain caused by HI. It was the first time that [NH_3_(CH_2_)_4_NH_3_]PbI_4_ was shown to be an alternate absorber, despite the fact that the PSCs only managed to attain a low PCE of 1.1%. Large colloidal particles in the perovskite precursor have been observed to dissolve when acidic additives are present, changing the shape of halide perovskite [117]. Safdari et al. [139] created thick, homogeneous perovskite films with greater surface coverage on the substrate by adding HI to the precursor solution, as an example. In compared to the reference model without the additive, the device's performance was prolonged when HI was introduced because it made the perovskite more soluble and produced dense layers. The greatest performance material below moisture environments was BDAPbI_4_ (1.08% PCE), that was equal to MAPbI_3_ solar cells (2.1% PCE). Both MAPbI_3_ and BDAPbI_4_ solar cells had an equivalent absorbed photon-to-current PCE, but the 2D PVK had wider band gaps and less photoconductivity.

Acidic-natured molecule additions have been shown to have a significant impact on the DJ perovskite shape [140] by encouraging the breakup of huge PVK particles into smaller ones with a more homogeneous dispersion of particles. Yu et al. [141] used the (PDMA)(MA)_5_Pb_6_I_19_ precursor solution to add HI acids to study the nucleation and development phases of perovskite layers. After the addition of acid, which caused the colloids in suspension to progressively disperse, the amount of tiny colloidal particles increased with longer aging times. Colloids are the nucleation centers, and this fact significantly influenced the crystallization kinetics and form of the thin layers. The density of nucleation sites was eventually reduced to produce huge grains and well-covering layers. Large crystal grains frequently came with unfavorable pinholes since crystal development might also proceed vertically. The best device was made with *n* = 6 in DJ perovskite, producing a PCE of up to 11%, which is remarkable.

As a result, the complete crystallization of those old precursors with acid additions required a higher temperature and longer time [122, 142]. The aged precursor produced a superior morphology with bigger grains, higher crystallinity, and better texture thanks to its smaller, more uniform colloidal particles [41, 143].

### Tin (II) Fluoride Additive

Due to fluorine's small size and high electronegativity [17, 144], fluoride species can interact with DJ PVK to generate powerful ionic and intermolecular bonds [125, 145]. The beneficial chemical reaction between fluoride and DJ PVK allows for the passivation of crystal boundaries and surface defects [14]. Fluoride is frequently appeared on the surface of DJ perovskite when it is integrated into the film [1, 121], displaying surface hydrophobicity and shielding the DJ PVK from moisture erosion [79, 117, 146]. In order to decrease the inherent Sn-cation vacancies in DJ phases (BDA)FA_2_Sn_3_I_10_ perovskites, Li and colleagues initially employed Tin (II) fluoride (SnF_2_) [102]. Theoretical simulations show that the addition of SnF_2_ considerably increases the chemical potential of Sn and the energy needed to create Sn vacancies. Furthermore, (BDA)FA_2_Sn_3_I_10_ has a high degree of crystal symmetry, which leads to efficient carrier separation and absorption. They were able to attain a PCE up to 6.43%, that is significantly greater than that of the FASnI_3_ cell (4.20%), by taking advantage of outstanding film forming and carrier transport features. More significantly, compared to 3D FASnI_3_ devices, the (BDA)FA_2_Sn_3_I_10_ devices demonstrated greater resistance to oxidation, light, and humidity.

The quick oxidation of Sn^2+^ to Sn^4+^ in ambient air is one of the crucial reasons for the minimal PCE and bad long-term steadiness of tin DJ PSCs [38]. Therefore, reductive additives are included to lessen the oxidation process for improved device performance, such as the frequently utilized divalent tin halides and their additives [67] and hydrazine derivatives. In order to create novel materials of the type FASnI_3_ with SnF_2_ additive, Ke et al. [92] presented a novel kind of tin-based PVK absorber that combines [45] both ethylenediammonium (en) and FA. Studies show that the tin double halide (SnF_2_) protects the FASnI_3_ perovskite films by acting as both an antioxidant and a tin source compensator. The atmosphere steadiness and photoelectric characteristics of the tin-based PVK absorbers were also markedly enhanced by the insertion of the en cation into the architecture.

While F ion (as SnF_2_) is stoichiometrically injected into the ASnI_3_ DJ PVK to create an ASnI_3-*x*_F_*x*_ DJ perovskite film, the tolerance factor, *t*, rises as *x* boosts due to the decreased F ionic radius and boosts the phase steadiness of Sn-based DJ perovskite [76]. The use of fluoride complex as an adjuvant consequently increased the stability of Sn-based DJ PVK. SnF_2_ was added by Ke et al. [147] to the perovskite solution to regulate the shape of DJ FASnI_3_ films. Propylenediammonium (PN) and trimethylenediammonium (TN) may both be incorporated into FASnI_3_ perovskite while still maintaining its three-dimensional structure and offering improved film shape and optoelectronic capabilities. It has been shown that adding SnF_2_ lowers background carrier density and Sn defects by increasing the Sn potential and lowering the energy required for the creation of Sn vacancies in lead-free PVK. Ma et al. [148] discovered that adding SnF_2_ to the precursor solution may create a homogeneous, thick, amorphous layer over the polycrystalline perovskite layer that would successfully obstruct oxygen and moisture from the outside while also reducing ion transport inside the devices. The layered 2D perovskite was made by mixing an organic cationic salt called hexamethylenediamine diiodide (HDAI) with FA tin iodide (FASnI_3_) PSCs.

**Fig. 9:**
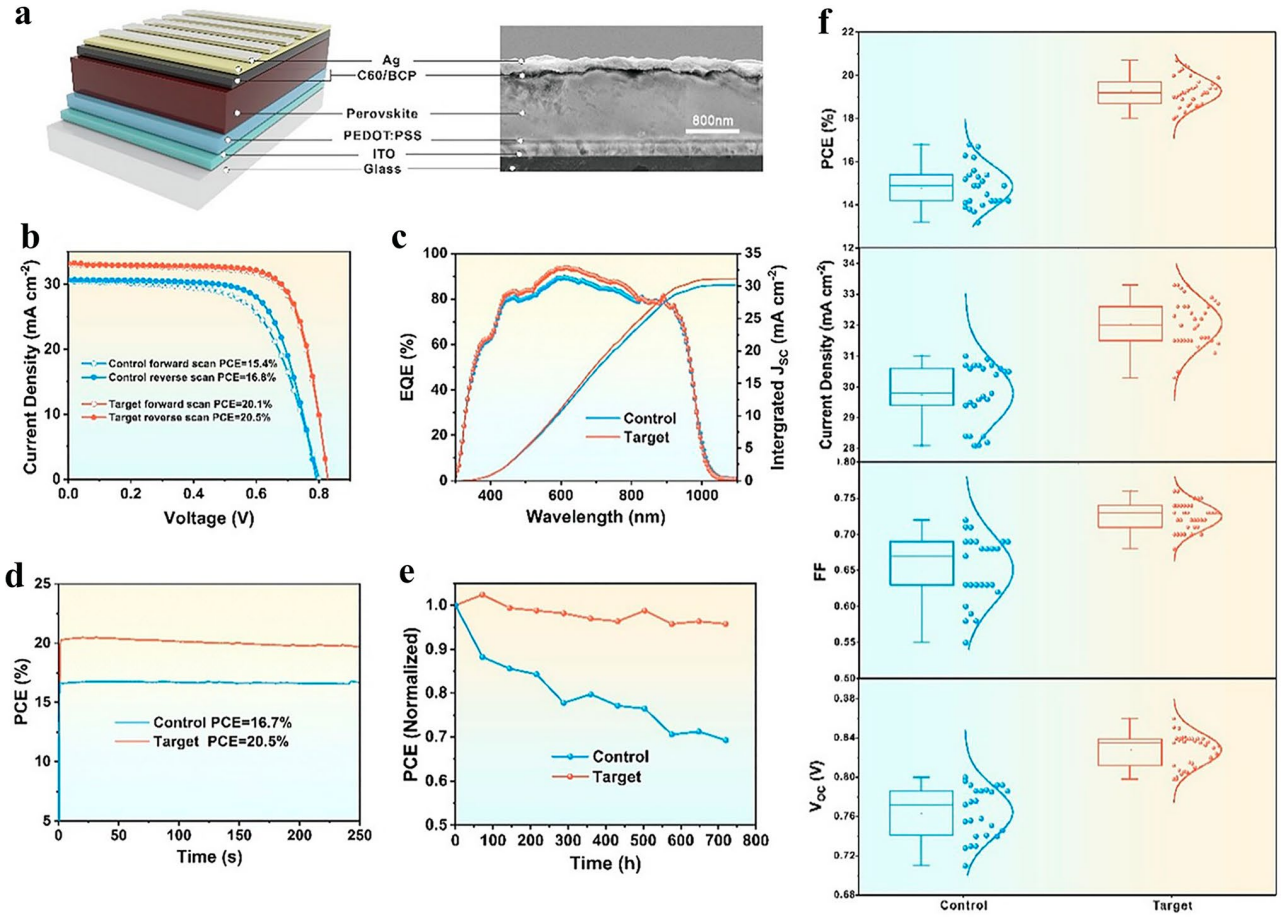
**a** The drawing of the PSC's inverted construction and cross-sectional SEM picture; **b** the control and target cell's current density–voltage curves in various scan directions; **c** the control and target cells' integrated Jsc curves and external quantum efficiency; **d** efficiency of control and target cells' steady-state outputs; **e** Stable testing of the control and target cells left unencapsulated in a glove box for more than 700 h; **f** PV parameter comparison between the control and target cells using statistics [150]. Copyright 2021, Wiley–VCH

Electron recombination can be decreased by interfacial engineering, the application of drugs in controlled dosages, or the adoption of HTL or ETL that may minimize cell recombination [24, 149]. When used as a single addition in DJ Sn-PSCs, SnF_2_ exhibits a superior response in this aspect [51]. According to Zhang et al. [150], the quasi-2D perovskite (PDMA) (FA_0.7_MA_0.3_)_3_(Pb_0.5_Sn_0.5_)_4_I_13_ (*n* = 4 phase) with SnF_2_ additive showed a blue-shifted absorption edge in Fig. 9 when compared to pure MASnI_3_ thin film. They established which the SnF_2_ additive represses the production of Sn^4+^ and significantly reduces the hole density (by 1–2 orders of magnitude). PSCs based on FA_0.7_MA_0.3_Pb_0.5_Sn_0.5_I_3_ films saw a considerable improvement in PCE and stability with the addition of a modest quantity of PDMADI. This method prevented Jsc deterioration, enhanced film crystallinity and shape, and allowed for a one order of magnitude decrease in defect density by not causing the bandgap to shift blue. The PSCs with 2D/3D bulk heterojunction Pb–Sn PVK finally achieved an enhanced efficiency of 20.5%.

SnF_2_ additive was utilized to improve phase stability [151]. In contrast to Pb^2+^, DJ PVK has a reduced bandgap when Sn^2+^ is used [65]; however, DJ PSCs exhibit instability and poor performance due to the phase change and rapid oxidation of Sn^2+^ to Sn^4+^ [152]. In order to make undoped (Sn^4+^ free) films with reduced defect concentration, Ke et al. [153] recommended that SnF_2_ simply function as an inhibitor of Sn^2+^ oxidation in the (3AMP)(MA_0.5_FA_0.5_)_*n*-1_(Pb_0.5_Sn_0.5_)_*n*_I_3*n*+1_ (*n* = 4) precursor solution. PVK films' shape was enhanced, and the crystallization process was changed using the SnF_2_ addition. The ionic radius of *F* is substantially less than that of I, hence F doping of iodide PVK won't cause any appreciable changes to the lattice parameter or undesirable film phases. As a consequence, solar cells utilizing 2D 3AMP and 3D perovskite composites as light absorbers were more efficient and stable, with a 20.09% power conversion efficiency. According to reports, tin fluoride (SnF_2_) protects the ASnX_3_ perovskite films by acting as both an antioxidant and a tin source compensator.

### Other Additives

**Fig. 10:**
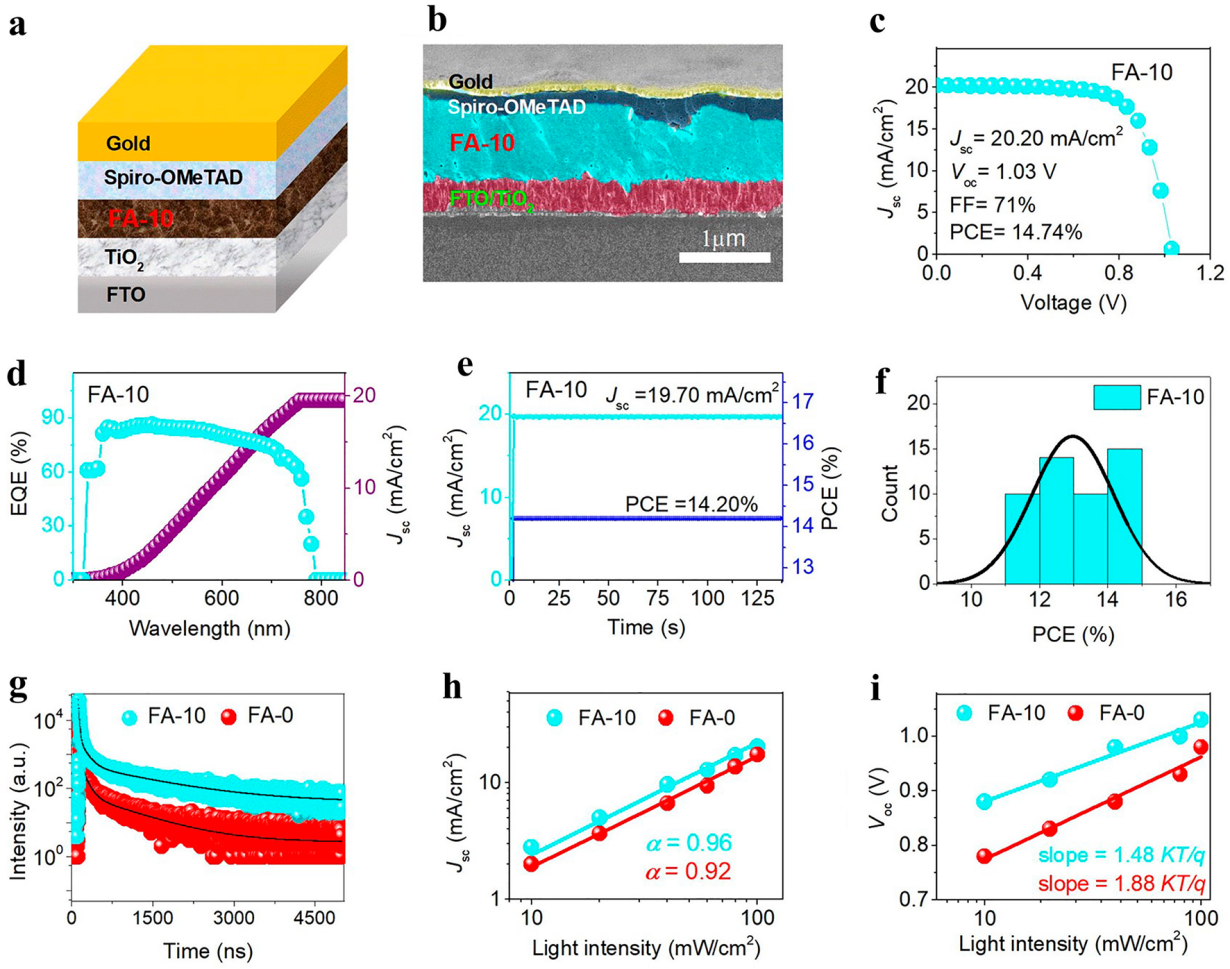
**a** Cell structure, **b** a SEM cross-sectional picture, **c** *J-V* curve, **d** EQE spectrum, **e** stabilized PCE and *J*_sc_, and **f** a histogram of PCEs collected from 50 FA-10-based DJ 2D PSC devices; **g** TRPL curves of films based on FA-0 and FA-10; **h** *J*_sc_ vs light intensity graphs that are double-logarithmic, and **i** *V*_OC_ vs light intensity semilogarithmic graphs for the FA-0 and FA-10-based cells [161]. Copyright 2021, Wiley–VCH

The functionality of DJ PSCs is considerably affected via the shape of perovskite films [154, 155]. The uniformity, coverage, and roughness of a surface are all influenced by the size and distribution of the grains, the texture, and pinholes in the films [156, 157]. The optimal situation is that PVK form a single crystal sheet on the substrate [105, 158, 159]. The nucleation, growth, and post-treatment stages of crystallization, as well as the coarsening or ripening process, can all be controlled to optimize the morphology [160]. For DJ perovskite films, additional chemicals, such as too much PbI_2_, are chosen and used. A surplus of formamidinium iodide can passivate the GBs (FAI). By creating self-assembled GBs on the ((PDA)(FA)_x_(MA)_3-x_Pb_4_I_13_ film, Ahmad et al. [161] chose the excess FAI to influence the grain surface of the PVK layers, resulting in better PVK performance and reduced hysteresis of PSCs in Fig. 10. DJ 2D perovskite with 10% FA^+^ doping has superior crystallinity, a preference for vertical orientation, and a lengthier charge carrier life than DJ 2D perovskite with no FA^+^. The 2D PSCs demonstrated an improved device stability and a champion PCE of 14.74%. After 6000 h of storing at a RH 65%, 800 h of exposure to warm at 85 °C, and 5000 h to one sun's light, the cells still hold more than 92%. These results imply that the DJ perovskite may be modified to produce extremely effective and steady DJ PSCs via the addition of FA cation.

Su et al. have disclosed [162] an additive method used for producing good DJ perovskite films devoid of MA, in which molecules of 1,1′-carbonyldi(1,2,4-triazole) (CDTA) are added to the PVK solution. Through CDTA adjustment, it is possible to change the distribution of phases, grain volume, crystallinity, crystal orientation, defect passivation. A further advantageous gradient phase distribution and subsequently gradient band alignment are produced, that is advantageous for carrier extraction and transport. The enhanced crystal orientation can make collecting and transporting carriers easier. As a result of the larger grain size and effective defect passivation, the trap density decreased. The result was a PCE of 16.07% from the CDTA-modified device. The cell retains 92% under irradiation and 86% of its original PCE after 360 h at 60 °C.

**Fig. 11:**
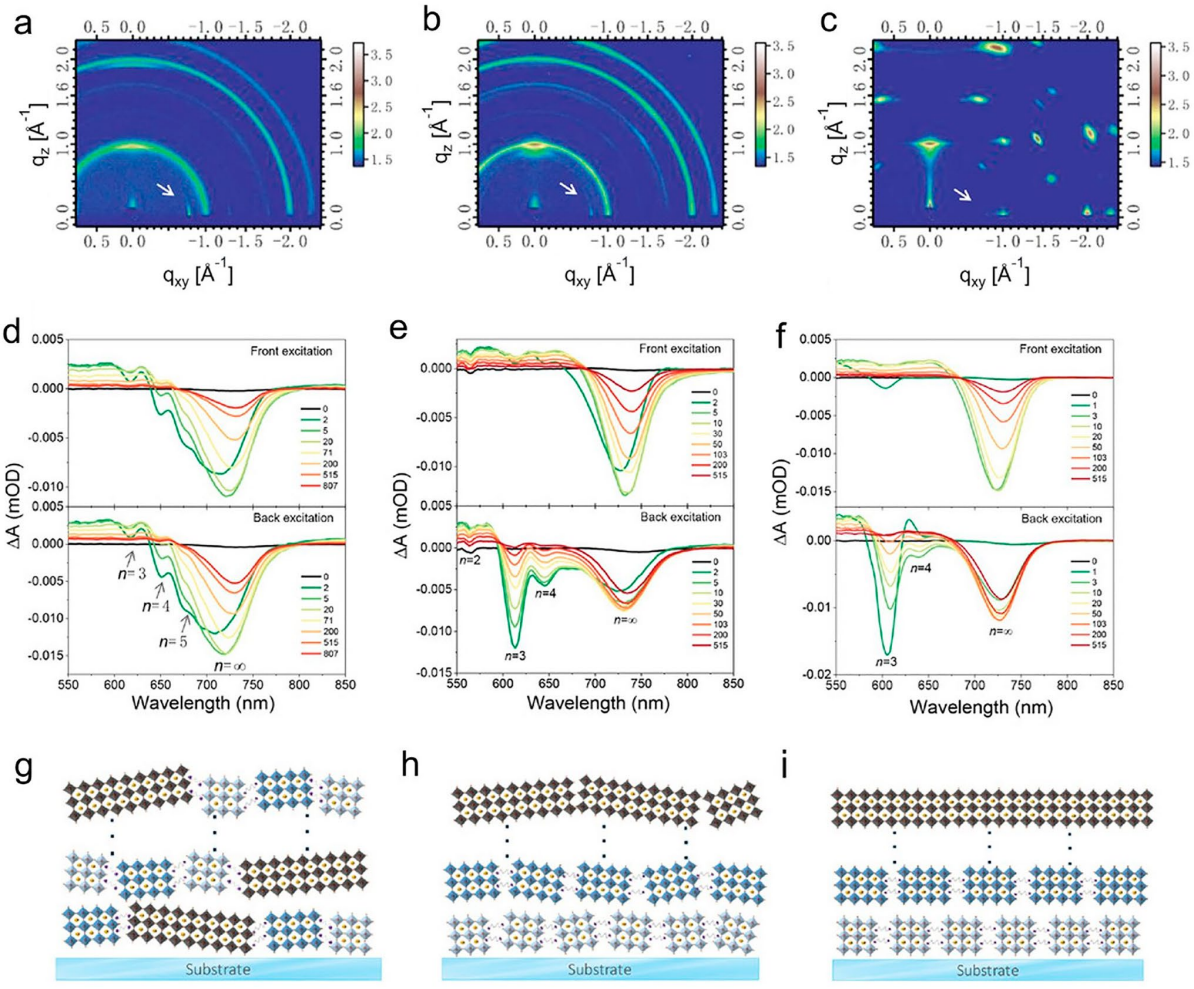
**a** The single-solvent DMF-deposited DJ multilayer perovskite films' GIWAXS patterns, **b** DMF:DMSO, a binary solvent and **c** DMF:DMSO:NMP, a ternary solvent **d** TA spectra of the DJ PVK layers formed from the single-solvent DMF at various delay durations, **e** DMF:DMSO, a binary solvent and **f** ternary-solvent DMF:DMSO:NMP; **g** DJ PVK with random crystal orientation, even phase distribution, and graded phase distribution are shown schematically with **h** random and **i** vertical crystal orientation [163]. Copyright 2022, ACS

Additionally, Chen [163] demonstrated in Fig. 11 the scalable printing of superior DJ perovskite thin films by adjusting crystallization kinetics. The standard DMF:DMSO-based precursor may be significantly delayed in crystallizing when a small quantity of 1-methyl-2-pyrrolidinone is added. This made it feasible for rapid interphase charge transfer. The winning perovskite cell generated a stable PCE of 16.19%. Additionally, thanks to their exceptional phase stability, the devices made with the ternary solvent displayed a noticeably improved stability when exposed to stimuli like light, heat, and humidity.

Due to its environmentally friendly, non-toxic, and superior photoelectric physical features, tin-based perovskite has received much research [103, 164]. The great sensitivity of Sn^2+^ cations to oxygen and moisture, however, posed a significant obstacle to creating robust solar cells [165]. Guo [166] discovered that the 4-(aminomethyl) pyridine (4AMPY) cation may be added to create the DJ perovskite 4AMPYSnX_4_. In the beginning, modulation of the halogen component improves charge transfer efficiency. The efficiency of charge transfer was further improved. In the end, they succeeded in fabricating good DJ PSCs with the best PCE of 5.03%. During 200 h of observing the functionality of unencapsulated devices in an ambient air (RH = 30%, *T* = 25 °C), there was no discernible PCE decline.

**Fig. 12:**
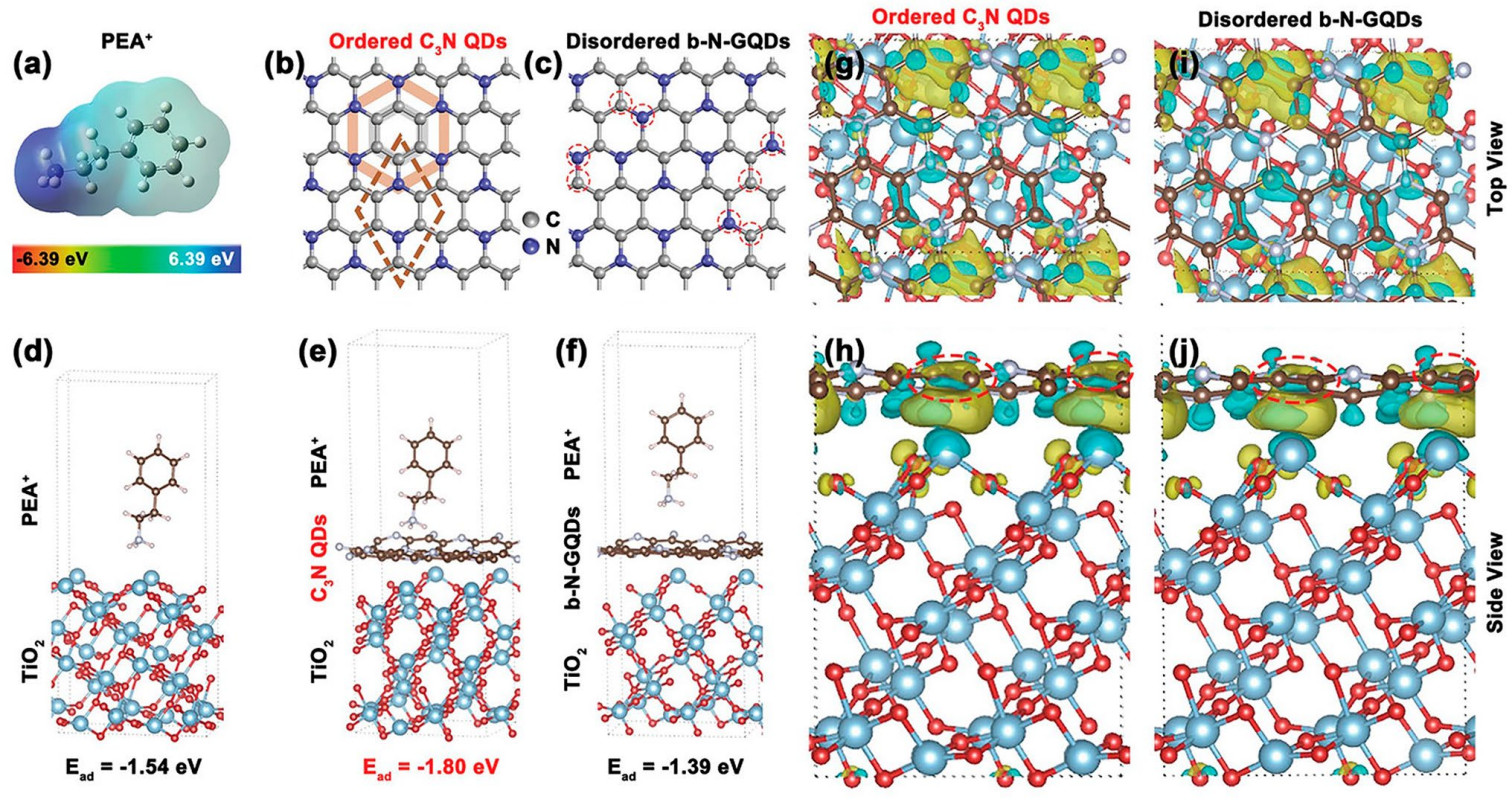
For the TiO_2_/perovskite surface's adsorption energy and charge redistribution, do the following DFT calculation: **a** PEA^+^'s electrostatic potential; **b** ordered C_3_N QD structure and **c** unorganized b-N-GQDs; **d** PEA^+^'s adsorption energy on TiO_2_ (101), **e** ordered C_3_N QDs and **f** disordered b-N-GQDs&TiO_2_ (101); views of the charge redistribution from above and from the side on **g, h** C_3_N QDs&TiO_2_ (101) and **i, j** b-N-GQDs&TiO_2_ (101) in the heterostructures [120]. Copyright 2022, Wiley–VCH

The fundamental problem preventing PSCs from being commercialized is volatility of performance under high RH [167], light exposure, and heat [104]. Lead halide perovskite quantum dots (QDs) [168, 169], that passivate the GBs and improve the crystallinity of the PVK [170], have been reported to improve the presentation of PSCs. To increase PCE of PSC devices, carbon quantum dots have proven to be effective perovskite absorber additions [171, 172]. Because of great charge carrier mobility and excellent transmittance over whole visible light spectrum, quantum dots are good-looking for photovoltaic devices. To control the crystallization of 2D PVSs, Li and colleagues [120] created a form of C_3_N QDs containing ordered carbon and nitrogen atoms. They caused the crystallization procedure, phase organization, and morphology to improve (Fig. 12). The QDs allowed for the creation of electron-rich regions to adsorb large organic cations and afford nucleation sites to achieve a bi-directional crystallization procedure. Combining theoretical modeling, morphological control, and femtosecond transient absorption characterization analysis allowed for this. Meanwhile, improved surface potential, compact topology, and decreased trap density all contributed to the improvement in 2D DJ film quality.

Salts called ionic liquids (Ils) are in liquid under 100 °C and are made up of massive organic cations and a variety of organic or inorganic anions. As a result of the strong interionic electrostatic force, they are practically nonvolatile. Normal IL properties include excellent thermal and electrochemical stability, as well as high ionic conductivity. A variety of chemical and inorganic substances can also be dissolved by ILs. By choosing various cation and anion combinations and permutations, you may make various binary and ternary ionic liquids. In newly developed quasi-2D-DJ phased PVK of (PDA)MA_4_Pb_5_I_16_ perovskite absorber, Ma et al. [173] used IL 1-butyl-3-methylimidazolium tetrafluoroborate (BMIMBF_4_) as an additive. The interaction between BMIM^+^ and perovskite also enabled a reduction in defect density, a suppression of ion motion, and improvements in film form and crystal quality. When the BMIMBF_4_ was introduced to the PVK, ion migration in the PVK layers was dramatically decreased. In order to prevent ions from migrating by intensifying chemical interactions in the vicinity, fluoride additive has only lately been utilized. In comparison with the control devices, which had an ideal PCE of 12.45%, the PSCs had a 14.07% PCE. Additionally, BMIMBF_4_ significantly increased the heat steadiness of the PSCs. The efficiency of the cells containing BMIMBF_4_ provides 72% of the original value after 120 h of heating at 85 °C, though efficiency of the control cells reduces to 19% of the initial value. The increase in performance was correlated with the benefits of employing IL BMIMBF_4_, including bigger grains, improved energy alignment at interfaces, and restricted ion migration.

Li and his colleagues [110] successfully developed the application of double organic ammonium salts with the same chain length (BAI and BDAI_2_). In contrast to using monoammonium BAI, they discovered that the BDA successfully decreases defect density and generates a 2D-DJ composition to improve interfacial charge extraction and prevent surface charge recombination. The outcome was an increased efficiency of 18.34%. It is common to attribute the enhanced stability to the massive organic cations that produce 2D constructions.

Ahmad [174] reported several DJ 2D perovskites that were cesium-doped (PDA = 1,3-propanediammonium) and explored the impact of cesium doping on characteristics and cell presentations. The 5% Cs-doped cells have a highest PCE of 18.30%, which is greater than the equivalent with no doping. Unencapsulated cells also show extraordinary stability, maintaining 95% of original efficiency after 5000 h of nonstop irradiation by one son, 240 h of moist heat at 85 °C and 95% RH, and 1000 h of MPP monitoring. The Cs doping's effects on improved film form, crystallographic orientation, lengthier charge carrier life, and decreased nonradiative recombination are responsible for the higher efficiency and stability. This study demonstrates that making extremely efficient and stable 2D PSCs is as easy and effective as adding Cs to DJ 2D perovskites.

**Fig. 13:**
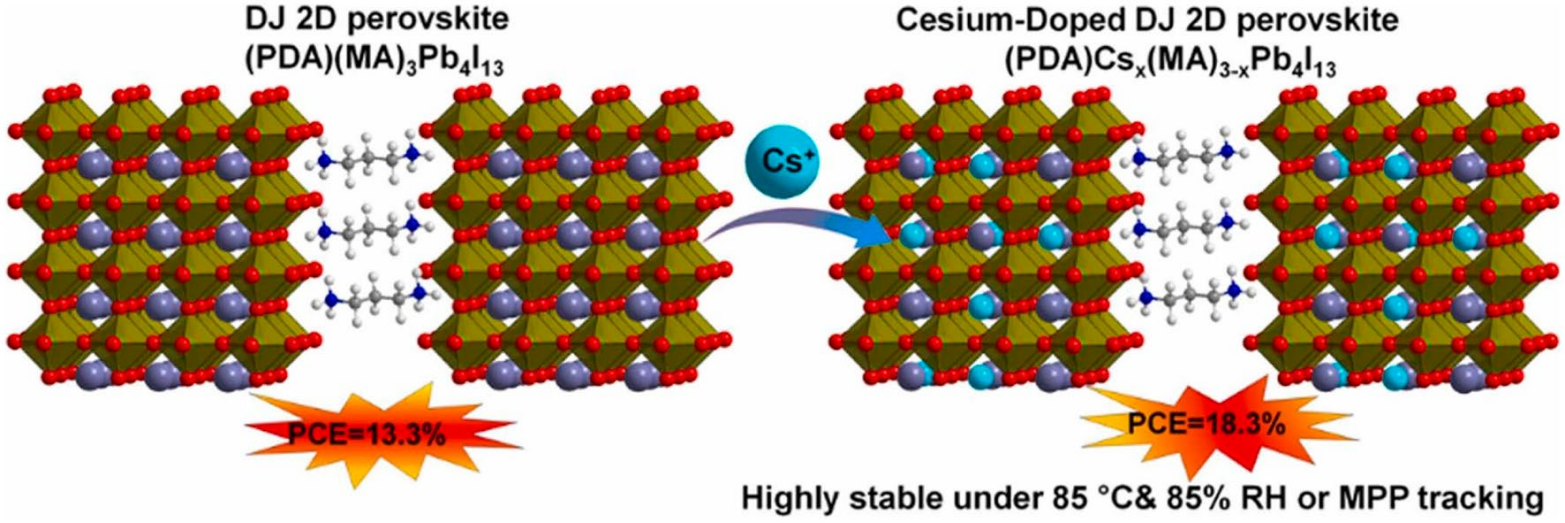
Illustration in schematic form of a Cs-doped DJ 2D perovskite [174]. Copyright 2022, Elsevier

Huang [175] learned the solvent function on layer generation and defect states of DJ PVK with methylammonium acetate (MAAc). It is studied that the nucleation procedure of DJ PVK can be blocked by extra coordination, that is evidenced by in situ optical spectra. As a result, out-of-plane oriented crystallization and ordered phase distribution are achieved. The thiourea (TU) [176] is positively utilized to make great efforts in optimizing the crystallization development, resulting in an increased grain size, flat and dense surface structure, and controlled delivery of n values for enhanced phase purity (Fig. 13).

These findings suggest that using additives is a viable method for creating DJ PSCs that are effective, stable, and free of hysteresis. There are differences between the introduction and existing forms of additives for DJ PSCs. Prior to the manufacture of the film, certain additives are added to the precursor, and others are added to the film after post-processing [127, 177]. After the creation of the devices, certain additives are eliminated while others are left in the finished products. For the remaining ones, some develop within the crystal, while others do so at edges, surfaces, or interfaces [110].

## Conclusion and Outlook

### Conclusion

We have shown that to produce effective, stable, and hysteresis-free 2D-DJ PSCs, additives have a great deal of promise and are frequently used. We have reviewed and described the various additives used for 2D-DJ PSCs. The geomorphology of perovskite layers is modulated via additives through changes in colloid size in the solution, formation of intermediates for nucleation, templating growing on substrates, causing coarsening of crystal size, and suppression of solution flow. This enables quick deposition techniques for more DJ perovskite layers. Additionally, nonradiative recombination may be avoided, ion migration can be suppressed, and DJ perovskite can be protected from harm by the coordination of additives by an ionic or other bonding at grain boundaries or on surfaces. This would boost V_OC_, eliminate hysteresis, and significantly enhance operating stability.

Three processes make up the DJ-phase multilayer perovskite crystallization process: (a) disordered colloidal sol–gel; (b) an oriented 3D-like phase; and (c) oriented 2D phase. The film-formation process of DJ-phase-layered perovskite is directly connected to the precursor in the first stage, which is also referred to as the intermediate stage. By creating intermediates between solvent molecules and PbI_2_, it is possible to prevent the quick crystallization of DJ-phase multilayer perovskite, which results in unpredictable orientation. The intermediate serves as a framework to make it easier to build perovskite QW because it progressively releases PbI_2_ to produce the perovskite phase as a result of the solvent evaporating during the annealing procedure. The creation of a 3D-like PVK phase at the gas–liquid interface, a crucial step, is subordinated in the second stage to the crystallization of the DJ-phase-layered PVK precursor. The desired orientation must be obtained with the constraint that the process is not interrupted. Additionally, whether preferred crystallization occurs inside the liquid or from the liquid–solid boundary, it is more likely to take on a random direction. Therefore, the secret to getting the desirable orientation in DJ-phase multilayer perovskites is managing the crystallization process. The DJ-phase-layered perovskite eventually expands and is preferentially orientated until the crystallization process is complete in the third step. In DJ-phase-layered perovskites, additive engineering can improve the nucleation and crystallization kinetics, resulting in the creation of crystals with well-defined shapes and sizes.

A fundamental concept is morphology. Making DJ perovskite precursor an actual solution would be intriguing for creating additive-assisted techniques for creating perovskite films that resemble single crystals or are highly oriented. Once the DJ film has attained a high level of quality and the grain boundaries are perpendicular to the base, it might be possible to develop additives to passivate the defects at the grain boundaries and shield them from deterioration by sturdy bonds for greater PCE and steadiness with no losing charge transport ability in one grain. Additionally, for those additives that are still found in DJ perovskite films, a detailed investigation of their effects on DJ perovskite energy level structure and energy level bending at interfaces is necessary to appreciate how these effects influence efficiency and hysteresis. DJ PSCs' device stability and efficiency may also be increased by adding the appropriate electrodes, interfacial modifiers, and charge-transport layers. Functional additives may be particularly created to passivate the faults in interfacial traps and DJ perovskite layers, which has shown to be a useful method to reduce energy loss and improve DJ PSC performance.

### Outlook

A few in situ characterization approaches are useful for completely understanding how the additives affect DJ perovskite crystallization, despite the fact that many groups have looked into the potential processes of additive-engineered DJ perovskite formation. To guide the creation of efficient additives, we must first get a fundamental understanding of how the different DJ perovskite materials' defect types and densities impact electrical transport properties. It is important to carefully examine the impacts and capabilities of additives with different molecular constructions. Consistent characterization methods should also be developed in order to precisely examine how additives affect the performance and stability of DJ PSCs. The development of a molecular library may result from research into how functional groups, conjugated systems, and extra substituents affect the passivation capacity. Once research data has been collected and analyzed, machine learning may be used to determine the fundamental concepts for selecting materials and improving technologies. The development of a molecular library might possibly result from research into the effects of functional groups, conjugated systems, and the extra substituent on the passivation capacity. Once research data has been collected and shared throughout the whole research community, we may apply machine learning to find the basic theories for choosing materials and improving equipment.

Although great advances in DJ perovskite have been made in recent years by using additive compounds, based on their original physicochemical and chemical advantages, numerous challenges such as a complex preparation process, low reproducibility, and toxicological safety remain for commercial application.

Although some challenges remain, additive compounds have increasingly been shown to be capable of preparing and improving DJ PSCs. A large variety of other optoelectronic devices, for example light-emitting diodes (LED), transistors, photodetectors, and lasers, as well as PSCs, still need to be constructed utilizing 2D DJ perovskites. A lengthy operational lifespan under ambient circumstances is envisaged in the future. Due to the 2D DJ perovskites' unique charge-transport capabilities along the in-plane and out-of-plane directions, careful examination in this area is necessary to further research into a variety of optoelectronic applications based on these materials.

The performance and stability of DJ perovskites can be enhanced using additive engineering, which is becoming more and more clear as a technique. The evaluation is anticipated to spur more efforts to support the usage of additive compounds in high-performing DJ perovskite systems, which can be applied for outstanding optoelectronic devices. The review is expected to pave the way for these key areas and stimulate more research to promote the use of additive engineering in excellent DJ perovskite optoelectronic device systems.
